# Burden, trends, and risk factors for breast cancer in China from 1990 to 2019 and its predictions until 2034: an up-to-date overview and comparison with those in Japan and South Korea

**DOI:** 10.1186/s12885-022-09923-4

**Published:** 2022-07-29

**Authors:** Na Liu, Da-Wei Yang, Yan-Xia Wu, Wen-Qiong Xue, Dan-Hua Li, Jiang-Bo Zhang, Yong-Qiao He, Wei-Hua Jia

**Affiliations:** 1 Department of Oncology, Luohe Central Hospital, Luohe, 462000 China; 2 State Key Laboratory of Oncology in South China, Collaborative Innovation Center for Cancer Medicine, Guangdong Key Laboratory of Nasopharyngeal Carcinoma Diagnosis and Therapy, Sun Yat-Sen University Cancer Center, Guangzhou, 510060 China

**Keywords:** Breast cancer, Trend, Prediction, Risk factor

## Abstract

**Background:**

The difference in epidemiological characteristics of breast cancer (BC) across countries is valuable for BC management and prevention. The study evaluated the up-to-date burden, trends, and risk factors of BC in China, Japan and South Korea during 1990–2019 and predicted the BC burden until 2034.

**Methods:**

Data on incident cases, deaths, disability-adjusted life-years (DALYs) and age-standardized rate (ASR) of BC were extracted from the Global Burden of Disease Study 2019. Trend analysis and prediction until 2034 were conducted by estimated annual percentage change and a Bayesian age-period-cohort model, respectively. Besides, the attributable burden to BC risk factors was also estimated.

**Results:**

In 2019, the number of BC incident cases, deaths and DALYs in China were 375,484, 96,306 and 2,957,453, respectively. The ASR of incidence increased, while that of death and DALYs decreased for Chinese females and Japanese and South Korean males during 1990–2019. High body-mass-index (BMI) was the largest contributor to Chinese female BC deaths and DALYs, while alcohol use was the greatest risk factor for Japanese and South Korean as well as Chinese males. The incident cases and deaths were expected to continue increase during 2020–2034 (except for Japanese female incident cases).

**Conclusions:**

China had the greatest burden of BC among the three countries. Incident cases and deaths of BC were projected to increase over the next 15 years in China, particularly among Chinese males. Effective prevention and management strategies are urgently necessary for BC control in China.

**Supplementary Information:**

The online version contains supplementary material available at 10.1186/s12885-022-09923-4.

## Background

Breast cancer (BC) is the first leading cause of cancer incidence and the fifth leading cause of cancer mortality worldwide in women, accounting for almost 1 in 4 cancer cases and for 1 in 6 cancer deaths [1]. Although BC in men accounts for approximately 1% of all BCs, the burden of male BC cannot be ignored and its incidence is rising year by year [2, 3]. Genetic and non-genetic risk factors associated with BC have been investigated extensively [4–6]. For example, mutations in BRCA1 and BRCA2 have a high risk of BC in women with odds ratios of 7.62 and 5.23 [7]. Multiple non-genetic risk factors include age, lifestyle (alcohol consumption, physical inactivity, tobacco smoking), anthropometric (high body mass index (HBMI)), metabolic syndrome, radiation exposure, sleep problems and negative emotions (NEs, depression, anxiety, psychosis, and psychological factors), while reproductive factors (early menarche, late menopause, low parity, shorter breastfeeding periods, late age at first birth and use of menopausal hormone therapy) only apply to female BC [4–6]. The incidence of BC varies due to geographic and temporal variations in BC risk factors [8]. Therefore, it is important to be aware of the latest BC burden and develop tailored prevention strategies at national level.

According to GLOBOCAN 2020, BC incidence rates are 88% higher in transitioned countries than in transitioning countries [1]. Although China is a transitioning country, the BC incidence rate is the highest of all cancers in Chinese females in 2020, reaching 37.7 per 100,000 [9]. Previous studies have revealed significantly increased effects of age and period and decreased effect of cohort on Chinese female BC incidence rates and Japanese and Korean mortality risks [10–12]. However, age, recent periods (2010–2015), and risk factor HBMI showed significant positive effect on death rates and disability-adjusted life-years (DALYs) among Chinese women BC in another study [13]. With rapid economic development, aging and growth of the population, the burden of BC in China may continue to grow in the future. On the other hand, Chinese male BC has been less studied. As a populated developing country in East Asia and the Pacific, China share similar genetic backgrounds and cultures with the developed Japan and South Korea. Therefore, assessment and comparison among these countries for female and male BC burden, trends, risk factors and projection of the future burden might provide valuable information for cancer control.

The Global Burden of Disease (GBD) study consists of a variety of epidemiological data on disease since 1990, which provides an unprecedented opportunity for comparable assessment of the BC burden, trends and risk factors among China, Japan and South Korea. In addition, the study used Bayesian age-period-cohort modeling and prediction (BAMP) models to project the BC burden among the three countries through 2034. The results might provide a scientific reference for BC control policy and help with effective allocation of healthcare resources for BC management and prevention in China as well as other developing countries.

## Methods

### Data sources

This study extracted data on the incident cases, deaths, disability-adjusted life-years (DALYs), as well as the corresponding age-standardized rates, in China, Japan, South Korea, East Asia and the Pacific region, and the world for the period of 1990–2019 from the Global Burden of Diseases (GBD) Study 2019 (http://ghdx.healthdata.org/gbd-results-tool) [14]. Detailed data sources and methodology of GBD 2019 were described in previous studies [15–17]. In brief, for China, the main data sources were Chinese Center for Disease Control and Prevention, National Central Cancer Registry, and Cancer Incidence in Five Continents (CI5). In Japan, the data was mainly from Vital Registration, Japan Center for Cancer Control and Information Services, Aichi Cancer Center Research Institute and CI5. Similarly, the main data sources for South Korea were Vital Registration, Korea Central Cancer Registry, Ministry of Health and Welfare, National Cancer Center and CI5. The International Classification of Diseases (ICD) version 9 and 10 codes pertaining to BC (ICD9: 174–175.9, V10.3, V16.3, and ICD10: C50-C50.629, C50.8-C50.929, Z12.3-Z12.39, Z80.3, Z85.3, Z86.000) were used to identify the cancer events. The socio-demographic index (SDI) is a composite indicator that reflects the socioeconomic development status of a country on a scale of the worst to the best, and its value ranges between 0 and 1 [15]. SDI values of the three countries were also from GBD 2019 [14].

### Estimation of attributable burden

The GBD 2019 estimate of attributable burden provides 560 risk-outcome pairs from published systematic reviews supported by credible or probable evidences. The comparative risk assessment (CRA) method applied in GBD 2019 was described in detail in previous literature [16]. Six risk factors were listed as paired outcomes of breast cancer in GBD 2019, including alcohol use (any gram per day of pure alcohol consumed among drinkers in a 12-month period), high body mass index (BMI) (defined as BMI greater than 20 to 25 kg/m^2^), high fasting plasma glucose (FPG) (any level above 4.8–5.4 mmol/L), low physical activity (< 3000–4500 metabolic equivalent (MET) minutes/week), diet high in red meat (any intake of red meat in 18 g per day including beef, pork, lamb, and goat but excluding poultry, fish, eggs, and all processed meat) and tobacco (smoking and chewing tobacco at level above zero and secondhand smoke exposure). We extracted data on the percentage of BC deaths and DALYs due to these six risk factors for the three countries from GBD 2019 [14].

### Study population

To predict the incidence and mortality of BC in the three countries, population estimates for 1990–2019 were obtained from GBD 2019 [14], while population projections for 2020–2034 were obtained from the United Nations Department of Economic and Social Affairs/Population Division [18]. Population data was stratified by year, sex (both, female, male) and age (18 age groups, from < 5 years to ≥ 85 years every 5 years). With respect to the projection period, probabilistic statistical techniques or general guidelines were used to determine the paths that fertility, mortality and international migration are expected to follow in the future. We used the medium-variant projections in this study.

### Model selection

Previously, some models have been used for cancer burden prediction, such as models based on three different time scales, age, period, and cohort (for example, age-period-cohort (APC) model [19], Bayesian age-period-cohort (BAPC) model fitted with Integrated Nested Laplace Approximation [20], Nordpred model [21], Bayesian age-period-cohort modeling and prediction (BAMP) model [22]), exponential regression (ER) model based on gross domestic product (GDP) per capita and time in years [23], approach assuming constant rates [24]. Some studies have demonstrated BAPC model fitted with Integrated Nested Laplace Approximation had the best predictive performance [20], while some studies have displayed ER model provided the best fit in almost all age-sex-cohort groups [23]. Lei and colleges have projected the burden of breast cancer in Chinese women by using the BAMP model with Gaussian random walk (RW) priors of different orders [22]. To select a model with the best predictive performance, we conducted a model comparison study. The BAPC model fitted with Integrated Nested Laplace Approximation, ER model, approach assuming constant rates and BAMP model with Gaussian RM1 and RM2 prior were trained for internal validation by using the GBD data. Incident cases and deaths of BC among women in China, Japan and South Korea were split into training set (data between 1990 and 2011) and testing set (data between 2012 and 2019), which were used to train and test the predictive models, respectively. Constant rates were calculated as the mean of observed rates of 2010 and 2011. The root-mean-squared error (RMSE) was applied to assess predictive performance. The RMSE was calculated as $RMSE=\sqrt{\frac{1}{N}\sum_{t=1}^{N}({Y}_{t}-\widehat{{Y}_{t}})}$, where ${\widehat{Y}}_{t}$ and ${Y}_{t}$ denote the prediction values and the observational values, respectively. Because the BAMP model with RW1 prior had the relatively lower RMSE (Table S1), we used it for the projection of BC case counts and rates through 2034.

The BAMP model has been described previously [22]. In short, the classical APC model can be formulated as ${\eta }_{ij}=l\mathrm{og}\left(\frac{{p}_{ij}}{1-{p}_{ij}}\right)=\mu +{\theta }_{i}+{\phi }_{j}+{\psi }_{k}$, Here ${\eta }_{ij}$ is the known population size of age group *i* at period *j* and ${p}_{ij}$ is the unknown incidence probability. The logit of the incidence probability is decomposed in an intercept µ, age effect${\theta }_{i}$, period effect ${\phi }_{j}$ and cohort effect${\psi }_{k}$. However, the APC model often has the problem of non-identifiability due to the untenable assumptions on parameter constrains. The BAMP model with Gaussian RW priors is less dependent on assumptions on parameters [25]. The RW1 prior assumes a constant trend over the time scale, whereas the RW2 prior assumes a linear time trend. In our study, a Gamma distribution with parameters a = 1 and b = 0.05 for overdispersion were used in BAMP model with RW1 prior.

### Statistical analyses

The age distribution of the world population from the GBD 2019 study was used to standardize rates of incidence, death, and DALYs per 100,000 person-years of BC (Table S2) [17]. The estimated annual percentage change (EAPC) was used to measure the temporal trend of observed and predicted values during 1990–2019 and 2020–2039. The natural logarithm of the value as a dependent variable was fitted with the calendar year using a linear regression model, ln(R) = α + β*calendar year + Ɛ, where R is number or rate. The EAPC was calculated as 100*(exp(β)-1), and the 95% confidence interval (CI) was obtained from the linear model [20]. The trend was increasing if the lower boundary of its 95% CI was > 0 and the trend was decreasing if the upper boundary of its 95% CI was < 0. Otherwise, the trend was regarded as stable over time [20]. A Pearson correlation test was conducted to assess the relation of the age-standardized rate (ASR) and SDI in the world and three countries from 1990 to 2019. We also divided the predicted BC incident cases and deaths into contributions from changes in risk, population size and age structure, according to methods used by Cheng et al. [26].

All analyses were performed using the statistical software R (Version 4.0.3; R Foundation for Statistical Computing, Vienna, Austria); R packages included ggplot2, RColorBrewer, BAMP, BAPC and INLA. A two-sided *P* value less than 0.05 was considered statistically significant.

## Results

### BC burden in 2019 and comparison with Japan and South Korea

In 2019, China had the largest number of incident cases (375,484), deaths (96,306) and DALYs lost (2,957,453) of BC, accounting for 58.27%, 49.82%, and 48.83% respectively, of the BC burden in East Asia and the Pacific and 18.75%, 13.75%, and 14.34% respectively, of the global BC burden. In addition, the number of incident cases, deaths and DALYs in China were 5.03, 6.0 and 7.44 times higher than those in Japan, and 18.47, 23.55 and 24.05 times higher than those in South Korea (Table 1 and Fig S1).

**Table 1:** The number of breast cancer case in the world, East Asia and Pacific, China, Japan and South Korea from 1990 to 2019

Variables	Number of Incident cases ^*^10^3^		Change, %	Number of Deaths ^*^10^3^		Change, %	Number of DALYs ^*^10^3^		Change, %
	1990 (95%UI)	2019 (95%UI)	1990–2019	1990 (95%UI)	2019 (95%UI)	1990–2019	1990 (95%UI)	2019 (95%UI)	1990–2019
World									
Both	876.99 (849.69–903.82)	2002.35 (1832.15–2172.54)	128.32	380.91 (364.81–396.71)	700.66 (647.38–751.56)	83.94	11,681.06 (11,169.25–12,261.81)	20,625.31 (19,043.05–22,174.4)	76.57
Female	867.62 (840.4–894.76)	1977.21 (1807.61–2145.21)	127.89	375.02 (358.98–390.82)	688.56 (635.32–739.57)	83.61	11,526.68 (11,021.13–12,107.83)	20,310.19 (18,744.8–21,866.65)	76.2
Male	9.37(8.81–9.96)	25.14(22.23–27.79)	168.3	5.89(5.43–6.37)	12.1(10.69–13.32)	105.43	154.37(142.7–166.95)	315.13(278.55–349.29)	104.14
East Asia and Pacific									
Both	176.37 (159.54–194.16)	644.39 (553.25–745.24)	265.36	85.73 (77.52–94.56)	193.31 (171.19–217.88)	125.49	2977.81 (2681.04–3311.9)	6056.26 (5369.26–6817.06)	103.38
Female	175.09 (158.27–192.94)	635.42 (543.84–737.37)	262.91	84.85 (76.68–93.69)	189.46 (167.25–214.31)	123.29	2952.93 (2657.26–3287.66)	5949.14 (5266.42–6720.58)	101.47
Male	1.27(1.12–1.44)	8.96(7.19–10.88)	605.51	0.88(0.77–1)	3.85(3.19–4.64)	337.5	24.88(21.7–28.31)	106.7(87.36–128.52)	328.86
China									
Both	81.62(66.87–97.1)	375.48(296.63–469.98)	360.03	41.8(34.55–49.51)	96.31(77.32–118.09)	130.41	1435.1(1184.14–1708.68)	2957.45(2408.51–3590.17)	106.08
Female	81.07(66.34–96.52)	368.37(290.09–463.34)	354.39	41.43(34.15–49.15)	93.5(74.51–115.42)	125.68	1423.49(1173.66–1696)	2877.24(2323.69–3513.54)	102.13
Male	0.55(0.45–0.66)	7.11(5.34–9.08)	1192.73	0.37(0.3–0.45)	2.81(2.15–3.53)	659.46	11.62(9.46–14)	79.97(61.25–100.58)	588.21
Japan									
Both	31.62(29.71–33.64)	74.6(59.58–91.31)	135.93	7.95(7.6–8.15)	16.04(13.49–17.43)	101.76	261.2(250.8–272.73)	397.77(358.07–434.85)	52.29
Female	31.48(29.57–33.51)	74.26(59.29–90.98)	135.9	7.89(7.54–8.09)	15.91(13.37–17.29)	101.65	259.69(249.31–271.17)	395.29(355.85–432.12)	52.22
Male	0.14(0.12–0.16)	0.34(0.26–0.43)	142.86	0.06(0.06–0.06)	0.13(0.11–0.14)	116.67	1.51(1.45–1.58)	2.48(2.25–2.71)	64.24
South Korea									
Both	3.46(3.24–3.72)	20.33(16.42–24.53)	487.57	1.43(1.35–1.51)	4.09(3.6–4.58)	186.01	51.58(48.82–54.68)	122.96(108.64–138.7)	138.39
Female	3.44(3.22–3.7)	20.25(16.36–24.45)	488.66	1.42(1.34–1.5)	4.06(3.58–4.55)	185.92	51.28(48.51–54.4)	122.29(107.94–138.06)	138.48
Male	0.02(0.01–0.02)	0.08(0.05–0.1)	300	0.01(0.01–0.01)	0.03(0.02–0.04)	200	0.3(0.26–0.35)	0.67(0.52–0.86)	123.33

*DALYs* disability-adjusted life years, *95% UI* 95% uncertainty interval

Among China, Japan and South Korea in 2019, the ASIR (31.02), ASDR (4.82) and age-standardized DALY rates (172.96) per 100,000 for both sexes in Japan were highest, which were about 1.72, 1.01 and 1.20 times higher than the rates in China. Although South Korea had the lowest ASDR (4.69) and age-standardized DALYs rates (143.56) per 100,000 for both sexes, the ASIR (23.79) per 100,000 was higher than that of China (18.32) (Table S3).

### Sex and age distribution and comparison with Japan and South Korea

Significant disparities in the sex distribution of BC were observed in the counts of incidence, mortality, and DALYs and the corresponding ASR. In 2019, 98.11% of incident cases (368,374), 97.08% of deaths (368,374) and 97.29% of DALYs (2,877,240) occurred in Chinese women, compared with 1.89%, 2.92% and 2.71%, respectively, in Chinese men (Table 1). The ASIR (35.61), ASDR (9.02) and age-standardized DALYs rate (277.98) per 100,000 in Chinese women were about 50.61, 30.10 and 34.96 times higher than those in Chinese men, respectively, which was the lowest among China, Japan and South Korea (251.96, 126.75 and 177.01, respectively in Japan and 220.95, 109.13, and 158.86 in South Korea) (Table S3 and Fig S2).

Due to the importance of age distribution in cancer epidemiology, patients were divided into three groups (15–49 years, 50–69 years, and 70 + years). The highest numbers of incident cases, deaths and DALYs were observed in patients aged 50–69 years, while the majority of age specific rates of incidence, death and DALYs peaked in the age 70 + years group. An exception was that the age specific rates of DALYs in Japan and China were slightly higher in the age 50–69 years group than in the 70 + age group (Figs S3 and S4).

### Trends in China and comparison with Japan and South Korea

Between 1990 and 2019, the number of the incident cases, deaths and DALYs of female and male BC increased greatly in China, Japan, South Korea and the world. The largest increase in female BC occurred in South Korea (488.66%, 185.92% and 138.48%, respectively), while China showed the greatest rise in male BC (1192.73%, 659.46% and 588.21%, respectively) (Table 1 and Fig S1).

Upward trends in the ASIR of BC were observed in China, Japan and South Korea. The ASIR increase in both sexes in China more than doubled (EAPC = 2.8; 95% CI: 2.70–2.91), whereas the increased speed of ASIR in males, with a rate of 8.37% (95% CI: 7.32%- 9.43%) per year, much higher than that in females of 2.66% (95% CI: 2.57%-2.76%) (Table S3; Figure S5). Among the three countries, the ASIR in the South Korean women (EAPC = 3.6; 95% CI: 3.27–3.94) had a fastest increase while the ASIR in men was stable (EAPC = 0.52; 95% CI: -0.04–1.09). Japan showed the lowest increase of ASIR in females (EAPC = 2.03; 95% CI: 1.8–2.27) and a medium increase of ASIR in males (EAPC = 0.65; 95% CI:0.36–0.93) (Table S3; Fig S5).

A marginally decreasing trend of ASDR (EAPC = 0.14, 95% CI: -0.21- -0.06) was observed in Chinese female BC, whereas a drastic increase was found in Chinese male BC (EAPC = 6.03, 95% CI: 5.08–6.97). On the contrary, a slight increase for females but a mild decrease for males of ASDR were observed in Japan and South Korea. The trends for ASR of DALYs in China, Japan and South Korea were similar to those for ASDR (Table S3; Fig S5).

### Association of ASIR, ASDR, and age-standardized DALYs rate with SDI

**Fig. 1:**
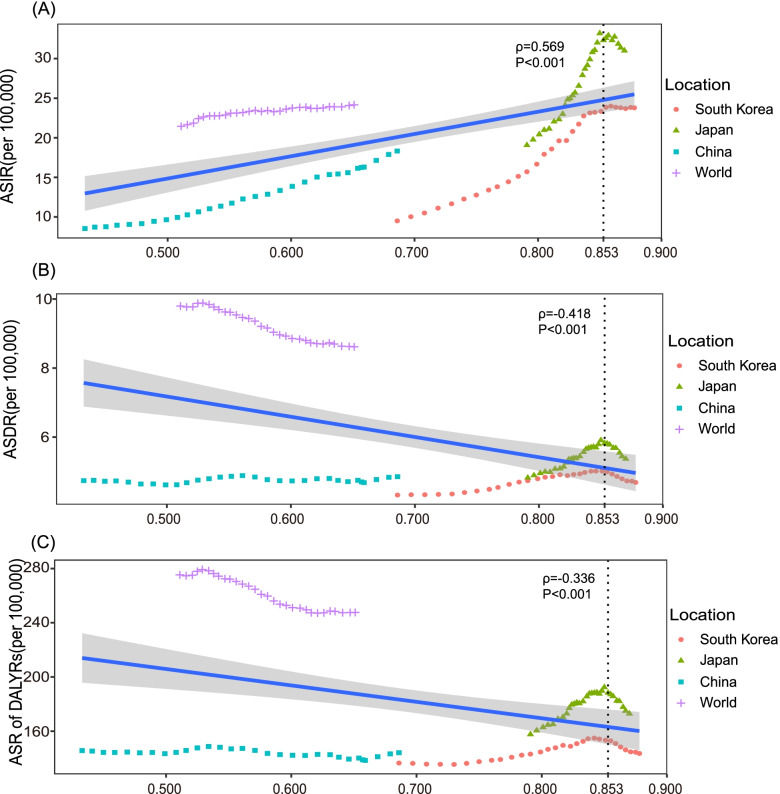
The trends in ASIR **A**, ASDR **B** and ASR of DALYs **C** of breast cancer in China, Japan, South Korea and the world by socio-demographic index for both sexes combined, 1990 to 2019. Expected values are shown as the blue line. ASIR, age-standardized incidence rate; ASDR, age-standardized death rate; ASR, age-standardized rate; DALYs, disability-adjusted life-years. The ρ was the Pearson correlation coefficient. The *P* values were derived from the Pearson correlation test

There was a positive association between ASIR in both sexes and the SDI value, however, an interesting phenomenon was that the ASIR decreased or remained stable in Japan (*ρ* = -0.80; 95% CI: -0.96- -0.23, *P* = 0.016) and South Korea (*ρ* = 0.30; 95% CI: -0.51- 0.83, *P* = 0.47) when the SDI value was greater than 0.853 (Fig. 1A). In contrast, a negative association was observed between ASDR of both sexes and the SDI value, whereas there existed a positive association when the SDI value was lower than 0.853 in China (*ρ* = 0.36; 95% CI:0–0.64, *P* = 0.048), Japan (*ρ* = 0.98; 95% CI:0.96–0.99, *P* < 0.001) and South Korea (*ρ* = 0.98; 95% CI:0.95–0.99, *P* < 0.001) (Fig. 1B). The association of the age-standardized DALY rate in both sexes with the SDI was similar to that of ASDR with the SDI (Fig. 1C). Meanwhile, the ASR in China and South Korea were below the expected value, while most of the ASR in Japan was above the expected value with the rise in the SDI value.

### BC death and DALYs attributable to risk factors

**Fig. 2:**
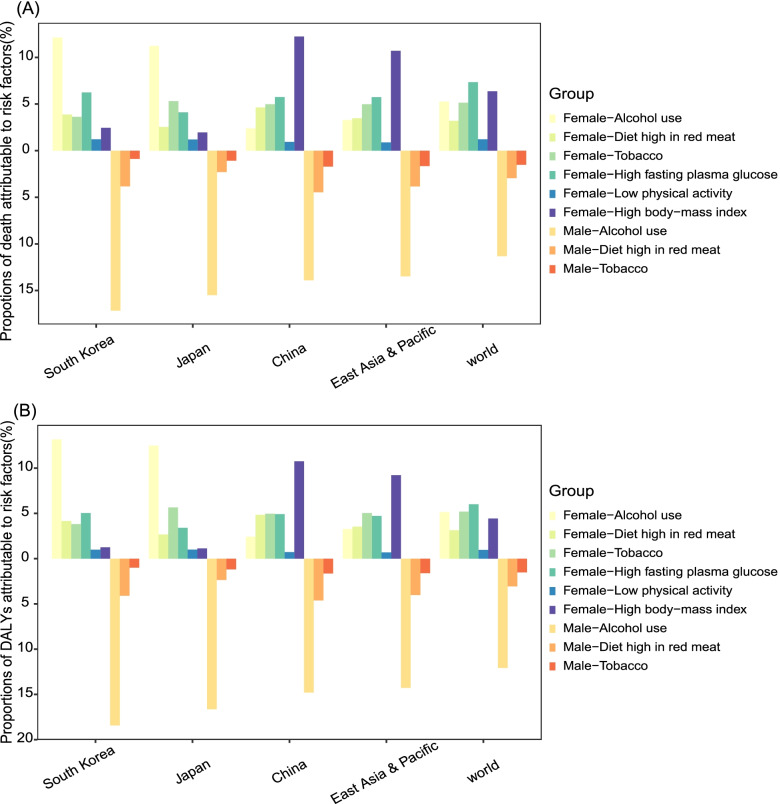
Proportions of Deaths **A** and DALYs **B** attributable to risk factors for female and male breast cancer in China, Japan, South Korea, East Asia and Pacific and the world in 2019. DALYs, disability-adjusted life-years

During 1990-2019, alcohol use was the largest contributor of BC deaths for females and males in Japan and South Korea, as well as for Chinese males, with the proportion of deaths ranging from 10.96% to 17.16% (Fig S6). For Chinese females, although alcohol use only led to 2.39% BC deaths in 2019, the proportion increased with a rate of 0.35% per year. The percentage of BC death attributable to high BMI was the highest for Chinese females and increased from 5.52% to 12.24%, with an EAPC of 2.99 (95% CI: 2.86-3.12). Meanwhile, Japan and South Korea also experienced increase in female BC deaths attributable to high BMI. High fasting plasma glucose (FPG) or tobacco were the second contributor of BC deaths for women in China, Japan and South Korea, while the deaths due to high FPG increased and those owing to tobacco decreased. Deaths associated with low activity, diet high in red meat and alcohol displayed different trends in China, Japan and South Korea (Fig 2A; Fig S6; Table S4). The percentages and trends of BC DALYs attributable to risk factors for females and males in the three countries were similar to those of BC deaths. (Fig 2B; Fig S7; Table S4).

### Predictions of BC incidence and death in China and comparison with Japan and South Korea

**Fig. 3:**
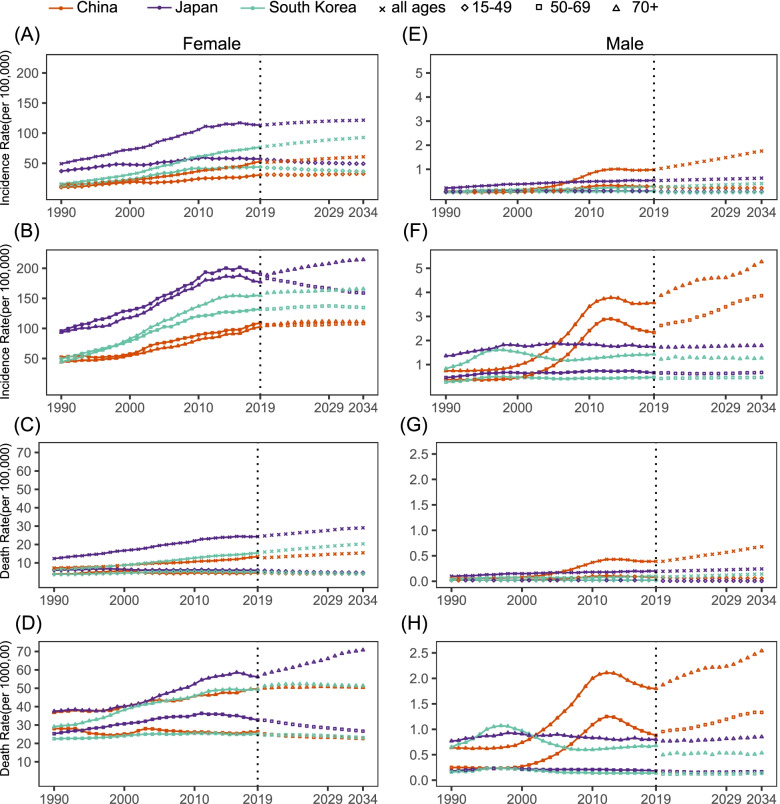
Trends in age-specific incidence and death rates during 1990–2019 and predictions from 2020 to 2034 in China, Japan and South Korea. **A**-**B** Female incidence; **C**-**D** Female death; **E**–**F** Male incidence; **H**-**I** Male death. The solid lines represent the observed value and the dots represent the estimated and predicted value calculated by Bayesian age-period-cohort modeling and prediction (BAMP) model; The vertical dashed grey line indicates year of 2019

**Fig. 4:**
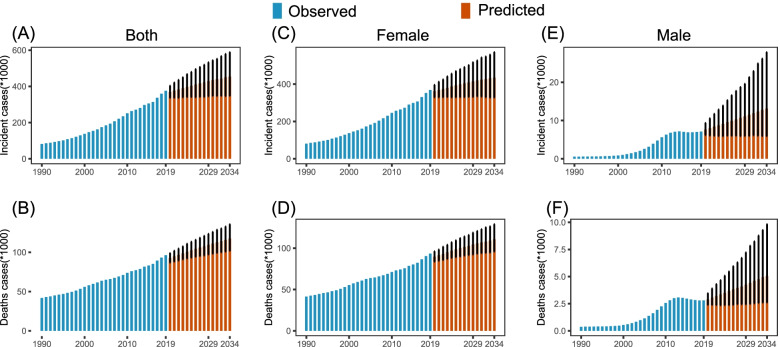
The number of incident cases and deaths during 1990–2019 and projected from 2020 to 2034 in China. **A**-**B** Both sexes; **C**-**D** Female; **E**–**F** Male. The error bar indicates 95% confidence interval (CI) of the number

We compared incidence and death rate calculated by BAPC model with observed rate from the GBD 2019 between 1990 and 2019, and found that they were nearly equivalent with each other (Fig S8, Fig. 3). For the period of 2020–2034, the ASIR of BC in women in China, Japan and South Korea were predicted to remain stable or decline, but the ASIR of BC in men in China and Japan would show an upward trend, particularly among Chinese men, with an annual increase rate of 1.66%, reaching 0.93/100,000 by 2034 (Table 2, Fig S8). The forecast incidence rate in Chinese and South Korean female and male breast cancers would continue to increase in most age groups, however, Japan would see the rise only in above 70 age group (Table 2, Fig. 3). The projected number of Chinese female incident cases in 2034 was 434,744, increased by 20.5% compared to 2020 with the changing age-specific incidence rates contributing 0.7%, population aging contributing 17.39%, and population growth contributing 2.41% to the total increase. Meanwhile, the number of Chinese male incident cases was 13,105, increased by 72.52% with changing age-specific incidence rates contributing 32.56%, population aging contributing 38.59%, and population growth contributing 1.37% to the total increase (Table S5, Fig. 4). Although the population of women and men in Japan declined (Table S6), predicted incident cases fell by 0.33% in women and increased by 10.21% in men due to different contributions from population aging and age-specific incidence rates compared to 2020 (Table S5, Fig S9). The number of new cases would be expected to continue to increase in South Korea (Table S5, Fig S10).

**Table 2:** The incidence rates of breast cancer in China, Japan and South Korea by sex and age, during 1990–2019, and projected until 2034

			Incidence Rate (/10^5^)		EAPC (95%CI)	
	1990 (95%UI)	2019 (95%UI)	2020 (95%CI)	2034 (95%CI)	1990–2019	2020–2034
China						
Both ^a^	8.54(7.07–10.11)	18.32(14.5–22.93)	18.21(15.28–21.75)	18.54(13.64–25.09)	2.8(2.7–2.91) ^*^	0.12(0.10–0.14) ^*^
Female ^a^	17.07(14.02–20.3)	35.61(28.07–44.81)	35.38(29.58–42.36)	35.47(25.89–48.48)	2.66(2.57–2.76) ^*^	0.02(0–0.04)
All ages	14.14(11.57–16.83)	52.81(41.59–66.43)	51.62(47.01–56.56)	60.67(45.78–79.71)	4.74(4.63–4.84) ^*^	1.17(1.12–1.23) ^*^
15–49	10.09(8.04–12.27)	30.41(23.18–38.57)	31.09(25.96–37.19)	32.52(23.80–44.75)	3.51(3.19–3.83) ^*^	0.43(0.28–0.59) ^*^
50–69	52.34(42.87–62.50)	109.19(85.72–137.57)	106.18(88.83–127.20)	108.11(78.69–146.95)	2.94(2.67–3.2) ^*^	0.18(0.11–0.24) ^*^
70 +	44.14(38.16–51.21)	102.62(83.91–125.27)	104.75(87.39–125.38)	110.89(80.71–151.48)	3.14(3–3.27) ^*^	0.34(0.18–0.5) ^*^
Male ^a^	0.13(0.1–0.15)	0.69(0.53–0.88)	0.74(0.57–0.95)	0.93(0.41–2.01)	8.37(7.32–9.43) ^*^	1.66(1.57–1.76) ^*^
All ages	0.09(0.07–0.11)	0.98(0.74–1.25)	1.03(0.84–1.27)	1.76(0.79–3.74)	11.03(9.89–12.16) ^*^	3.79(3.68–3.9) ^*^
15–49	0.04(0.03–0.04)	0.29(0.21–0.37)	0.26(0.19–0.34)	0.21(0.09–0.47)	9.54(8.38–10.69) ^*^	-1.27(-1.51–1.03) ^*^
50–69	0.37(0.29–0.45)	2.33(1.71–2.99)	2.62(2.02–3.39)	3.86(1.72–8.21)	9.23(8.02–10.44) ^*^	2.98(2.81–3.15) ^*^
70 +	0.74(0.6–0.9)	3.57(2.8–4.46)	3.87(2.94–5.06)	5.27(2.36–11.54)	7.39(6.56–8.21) ^*^	1.87(1.62–2.12) ^*^
Japan						
Both ^a^	19.07(17.92–20.29)	31.02(24.85–38.16)	30.59(28.12–33.30)	29.37(23.67–36.00)	1.94(1.71–2.18) ^*^	-0.30(-0.32–0.28) ^*^
Female ^a^	36.07(33.92–38.39)	60.47(48.35–74.56)	59.52(54.71–64.80)	56.57(45.74–69.41)	2.03(1.8–2.27) ^*^	-0.38(-0.4–0.36) ^*^
All ages	49.2(46.21–52.37)	113.34(90.5–138.86)	114.00(107.91–120.42)	121.56(99.72–147.39)	3.17(2.94–3.41) ^*^	0.45(0.39–0.5) ^*^
15–49	36.91(33.14–41.06)	57.11(44.16–73.46)	55.75(50.67–61.05)	49.16(39.36–60.79)	1.53(1.33–1.74) ^*^	-0.9(-1.03–0.76) ^*^
50–69	95.83(87.98–104.76)	190.13(148.94–237.62)	183.19(168.07–200.02)	158.94(128.55–195.23)	2.75(2.54–2.96) ^*^	-1.05(-1.1–1.01) ^*^
70 +	93.50(82.55–103.23)	177.24(130.93–218.90)	188.58(172.77–205.48)	214.22(173.59–262.68)	2.77(2.53–3.02) ^*^	0.91(0.83–0.99) ^*^
Male ^a^	0.19(0.17–0.22)	0.24(0.19–0.31)	0.23(0.19–0.28)	0.24(0.17–0.32)	0.65(0.36–0.93) ^*^	0.02(0–0.03) ^*^
All ages	0.22(0.19–0.25)	0.54(0.42–0.69)	0.53(0.46–0.62)	0.63(0.47–0.82)	2.89(2.53–3.25) ^*^	1.17(1.15–1.19) ^*^
15–49	0.07(0.05–0.09)	0.1(0.07–0.14)	0.07(0.02–0.19)	0.07(0.01–0.19)	1.25(1.01–1.49) ^*^	0.01(-0.41–0.43)
50–69	0.46(0.38–0.57)	0.66(0.46–0.94)	0.65(0.41–0.95)	0.67(0.4–1.03)	1.09(0.76–1.41) ^*^	0.25(-0.06–0.56)
70 +	1.36(1.13–1.67)	1.74(1.31–2.28)	1.72(1.2–2.33)	1.79(1.16–2.59)	0.66(0.37–0.95) ^*^	0.32(0.27–0.37)
South Korea						
Both ^a^	9.5(8.9–10.19)	23.79(19.29–28.65)	23.75(21.25–26.54)	23.07(16.67–31.25)	3.42(3.07–3.78) ^*^	-0.23(-0.25–0.20) ^*^
Female ^a^	17.5(16.39–18.81)	46.4(37.59–56.14)	46.10(41.24–51.67)	43.87(31.60–60.13)	3.6(3.27–3.94) ^*^	-0.36(-0.4–0.31) ^*^
All ages	15.61(14.61–16.78)	76.66(61.92–92.55)	78.29(71.90–85.240	92.76 (67.71–125.14)	5.78(5.41–6.15) ^*^	1.21(1.13–1.3) ^*^
15–49	12.22(11.06–13.61)	43.73(33.51–55.05)	42.57(37.35–48.40)	36.26(25.72–50.42)	4.7(4.19–5.21) ^*^	-1.19(-1.24–1.13) ^*^
50–69	49.38(44.63–54.80)	131.88(102.87–166.01)	132.04(117.30–148.47)	134.68(97.33–184.95)	3.56(3.26–3.87) ^*^	0.18(0.05–0.31) ^*^
70 +	44.46(39.68–49.94)	154.60(120.05–192.00)	158.99(140.40–180.13)	165.59(119.64–225.98)	4.65(4.24–5.05) ^*^	0.27(0.25–0.29) ^*^
Male ^a^	0.13(0.11–0.15)	0.21(0.15–0.28)	0.17(0.12–0.25)	0.17(0.09–0.3)	0.52(-0.04–1.09)	-0.07(-0.09–0.06) ^*^
All ages	0.07(0.06–0.09)	0.29(0.2–0.39)	0.26(0.19–0.35)	0.41(0.23–0.65)	3.86(3.39–4.32) ^*^	3.05(2.94–3.17) ^*^
15–49	0.04(0.03–0.05)	0.08(0.05–0.12)	0.04(0–0.16)	0.04(0–0.23)	2.08(1.66–2.51) ^*^	1.47(0.04–2.9) ^*^
50–69	0.27(0.2–0.37)	0.47(0.28–0.74)	0.42(0.16–0.81)	0.46(0.15–0.94)	0.79(0.24–1.34) ^*^	0.62(0.48–0.75) ^*^
70 +	0.83(0.58–1.16)	1.43(0.88–2.27)	1.22(0.41–2.4)	1.27(0.49–2.49)	0.54(-0.11–1.19) ^*^	-0.03(-0.3–0.25)

*EAPC* estimated annual percentage change, *95% UI* 95% uncertainty interval, *95% CI* 95% confidence interval

^a^ Age-standardized incidence rate

^*^ Changes that are statistically significant

The predictive ASDR of BC in the three countries between 2020 and 2034 decreased, with the exception of an annual increase of 1.55% in that of Chinese men (Table S7, Fig S8). The 15–49 age group would see the decrease in death rate while the people beyond 70 years of age would continue to remain the highest rate (Table S6, Fig. 3). However, the three countries were projected to experience a sustained rise in BC deaths (Table S5, Fig. 4, Figures S9-S10).

## Discussion

The present study undertook a comprehensive analysis and comparison of burden, temporal trend and risk factors for BC in China, Japan and South Korea from 1990 to 2019, and further predicted BC incidence and death data over the next 15 years. Our results demonstrated that the number of incident cases, deaths and DALYs of breast cancer in the three countries increased dramatically in the last 30 years, and the incident and death cases were projected to continue to rise between 2020 and 2034, except for a slight drop in Japanese female incident cases during 2026–2034. Compared with Japan and the Republic of Korea, China had a heavier burden of BC.

China had a lower ASIR of BC in both sexes than Japan and the South Korea, but all witnessed dramatic rises between 1990 and 2019, which could be explained by the heterogeneity in the prevalence of risk factors and mammography screening rates. We utilized SDI to reflect socioeconomic status. The ASIR increased as the SDI rise when the SDI value was lower than 0.853. The increase might be from the increasing socioeconomic level, westernized lifestyles [27–29], changes in reproductive pattern (low parity, a short duration of breastfeeding, late age at first birth) [30–32] and rising obesity rate [33, 34]. In addition, low blood vitamin D levels and sleep problems including light exposure at night, night/shift work, late sleeping, and frequent night waking were reported to increase breast cancer risk [35, 36]. The increased and high prevalence rate of vitamin D deficiency and sleep problems in China and South Korea may also contribute to the increase in ASIR [37–40]. The decline or stabilization in ASIR when the SDI value was higher than 0.853 in Japan and South Korea could be attributed to the plateaus in participation of mammography screening [41]. Compared with the nationwide breast cancer screening program in South Korea in the early 2000s [42], Cervical Cancer and Breast Cancer Screening Program targeting rural women aged 35–64 years in 2009 and Cancer Screening Programs in Urban Area in 2012 were lanced in China [43]. Moreover, the participation rate of 21.7% in China was lower than that of 44.9% in Japan [44]. The ASIR in female BC in the three countries were projected to remain stable or decrease whereas the crude rate increased during 2020–2034. Population aging was the main contributor and risk factors also played an important role according to our results. Besides, it has been reported that the program targeting cervical cancer and breast cancer will cover 80% and 90% of all county-level regions by 2022 and 2030 in China [43]. The predictive incident cases of Chinese female BC in 2030 and 2034 was 419,673 and 434,744, respectively, which is consistent with the estimate of 413,800 new cases in 2030 in another study [22]. To be noted, China witnessed a sharpest increase in male BC incident cases in the past three decades, which was predicted to increase significantly in the next 15 years. Recent study has revealed that negative emotions can increase the risk for the incidence of BC, and can be supportive of the prognosis of the disease [45], the increasing and high incidence of depression in the elderly population might be related to the male BC incident cases [46]. Our data supports that much more attention is required for the Chinese male category, despite the low incidence rate in male BC.

We found that despite the slight increase in ASDR of female BC in Japan and South Korea, the increase rates per year in ASDR were much lower than those in ASIR. Additionally, the ASDR in Chinese female BC declined. Several reasons could explain the situation. Firstly, the improvements and progress in treatment have been shown to increase overall survival [47]. For example, more patients received radiotherapy and endocrine therapy in China [48]. Growing new drugs such as anti-Her-2/neu antibody [49], selective estrogen receptor down-regulator [50] and programmed cell death ligand 1 inhibitors [51] have been applied. A study has reported that every 10% increase in the reimbursement rate is associated with a 7% reduced risk of cancer-specific mortality [52]. The substantial progress for China since 2009 [53] and the achievement for Japan and South Kora before 1990 [54, 55] in universal health insurance coverage were the second possible explanation. Thirdly, early detection by screening has higher survival rate. In addition, present precision medicine may contribute to the decline in predictive ASDR of BC in the three countries between 2020 and 2034. For examples, researches refined to specific molecular structures such as COL10A [56] and SPARC [57] may be meaning for clinical outcome and prognosis.

China, Japan and South Korea had different risk factor profiles of BC. Different from that high FPG was the leading cause of global BC deaths [8], high FPG was the second or third cause of BC deaths and DALYs for Chinese, Japanese and South Korean females. Similarly, an increasing contribution of high FPG was observed, which was in line with the rise in diabetes prevalence in the three countries [58–60]. Major established risk factors for diabetes included obesity, family history and westernized lifestyle [58–60]. The current research has demonstrated that the changes in the expression and secretory spectrum of inflammatory mediators of adipocytes and the increase in other inflammatory factors promotes the proliferation and invasion of tumor cells and the formation of neovascularization, especially in obese individuals [61]. Overweight and obesity have increased rapidly in China [33], steadily in Japan and South Korea [34, 62], which was consistent with the upward trend in high BMI related death and DALYs. However, the percentages of deaths and DALYs attributable to high BMI were higher in China than in Japan and South Korea, and highest in China, which was due to the high prevalence of overweight persons [63]. Sustained weight loss, even modest amounts could reduce the risk of breast cancer in women 50 years and older [64]. Therefore, it is urgent for the government to take a series of measures to combat the rising obesity and improve public awareness of diabetes and glycemic control. Physical activity attenuates breast cancer risk and improves survival [65, 66]. The potential mechanism could be that physical activity or exercise modulates macrophage anti-tumor activity [65]. Contrary to Japan and South Korea, a decreasing proportion of deaths and DALYs owing to low physical activity occurred in Chinese females. The fasting growing Chinese square dancing may partly explain the downtrend [67]. Alcohol consumption was positively associated with risk of BC. Potential mechanisms for the carcinogenesis of mammary tumors include DNA damage and gene mutation induced by acetaldehyde (a product of alcohol metabolism), altering circulating sex hormone levels [68]. In addition, alcohol enhances the aggressiveness and malignancy of breast cancer [68]. Alcohol was the leading contributor of BC deaths and DALYs for Japanese and South Korean populations, as well as for Chinese males. Fortunately, Japan and South Korea showed a decrease trend in alcohol related deaths and DALYs. With the rapid economic development and the well-developed traditional drinking culture, the increasing proportion of deaths and DALYs owing to alcohol for Chinese males and females was a particular concern. Our study indicated the deaths and ADLYs attributable to tobacco descended. Nevertheless, the prevalence of tobacco use is still high [69], which suggests that tobacco control still has significant implication for reducing the burden of BC in the future. The rising speed of deaths and DALYs attributable to diet high in red meat for females and males in China and South Korea, as well as for Japanese males was only second to high BMI. Recent study has confirmed that a higher healthy lifestyle index (HLI) score is associated with a lower BC risk and mortality [70]. HLI scores were generated from body mass index, physical activity, intake of plant and animal foods, alcohol consumption, breastfeeding, and smoking, with higher values corresponding to healthier behaviors [70]. Therefore, adopting healthy lifestyles should be given priority for cancer prevention.

Based on the GBD 2019 study, Xu and colleagues has reported burden and trends of BC from the macroscopic point of view [8]. However, our study specially analyzed the epidemiology of BC in China, Japan and South Korea and predicted the future burden, which could provide country-tailored guidance for the prevention and control strategies of BC. Lei et al. has evaluated the incidence and death trends and predict the burden of Chinese female BC by using the data from the National Central Cancer Registry [22]. The trends and prediction were almost consistent with our results. Furthermore, we also displayed the increasing burden in Chinese male BC and related risk factors.

Our study has some limitations. First, although GBD study incorporates methods to adjust for incomplete or missing data and quality of the data, there may still be the possibility of some inaccuracy in the mortality data. Second, this is an ecological study, the interpretations derived from this are true at population level but do not necessarily hold for individual level. Third, other risk factors related to BC are limited. Genetic factors such as intrinsic genes, estrogen and estrogen receptors and environmental factors such as ionizing radiation were not available on the online database. Finally, the two-child policy since 2015 and the three-child policy since 2021 in China and the COVID-19 pandemic since 2019 have had a great impact on the national population and medical resource allocation, which may influence the population projected by the United Nations. In addition, if the two-child and three-child policies can be implemented, the incidence rate of female BC may decline in the future.

## Conclusions

In conclusion, the counts of BC incident cases and deaths in China, Japan and South Korea increased rapidly during the past 30 years and were predicted to continue to rise during 2020–2034 (except for Japanese females), particularly among Chinese males, which remained a great burden for public health. High BMI and alcohol use were found as the largest modifiable risk factors for BC deaths in Chinese women and man, respectively. In view of reducing the burden of BC, the following recommendations should be put forward for women and men: avoid or reduce excess weight, limit alcohol consumption, engage in sufficiently intense and regular physical activity, avoid exposure to active or passive smoking and healthy diet. In addition, Chinese policymakers should consider improving public health awareness, especially amongst older women and establish screening programs suitable for the Chinese population. Meanwhile, the precise intervention, diagnosis and treatment of BC should be strengthened.

## Supplementary Information

**Additional file 1:** **Fig S1.** The number of breast cancer incident cases, deaths and disability-adjusted life years (DALYs) by sex and location from1990 to 2019. (A-C) Both sexes; (D-F) Female; (G-I) Male. **Fig S2.** The ASIR, ASDR, and ASR of DALYs of breast cancer in China, Japan, South Korea,East Asia and Pacific and the world from 1990 to 2019. (A-C) Both sexes; (D-F) Female; (G-I) Male. ASIR: age-standardized incidence rate, ASDR: age-standardized death rate, ASR:age-standardized rate, DALYs: disability-adjusted life-years. **Fig S3.** The number of breast cancer incident case, deaths and disability-adjusted life years (DALYs) for both sexes by age group in China, Japan, South Korea, East Asia and the Pacific and the world in 2019. The error bar indicates 95% uncertainty interval of the number. **Fig S4.** The rates of breast cancer incidence, death and disability-adjusted life years (DALYs) for both sexes by age group in China, Japan, South Korea, East Asia and the Pacific and the world in 2019. The error bar indicates 95% uncertainty interval of the rates. **Fig S5.** The EAPCs of ASIR, ASDR and ASR of DALYs for breast cancer from 1990 to 2019 by sex in the China, Japan, South Korea, East Asia and the Pacific and the world. EAPC: estimated annual percentage change; ASIR: age-standardized incidence rate; ASDR: age-standardized death rate; ASR: age-standardized rate; DALYs: disability-adjusted life-years. The error bar indicates 95% confidence interval (CI) of the EAPCs. **Fig S6.** Temporal trends of percent of deaths for breast cancer attributable to risk factors in China(A), Japan (B) and South Korea (C), 1990-2019.  **Fig S7.** Temporal trends of percent of DALYs for breast cancer attributable to risk factors in China(A), Japan (B) and South Korea (C), 1990-2019. DALYs: disability-adjusted life-years. **Fig S8.** Trends in ASIR and ASDR during 1990–2019 and predictions from 2020 to 2034 in China(A-B), Japan (C-D) and South Korea (E-F). ASIR: age-standardized incidence rate; ASDR: age-standardized death rate. **Fig S9.** The number of incident cases and deaths during 1990-2019 and projected from 2020 to 2034 in Japan. (A-B) Both sexes; (C-D) Female; (E-F) Male. The error bar indicates 95% confidence interval (CI) of the number. **Fig S10.** The number of incident cases and deaths during 1990-2019 and projected from 2020 to 2034 in Republic of Korea. (A-B) Both sexes; (C-D) Female; (E-F) Male. The error bar indicates 95% confidence interval (CI) of the number. **Table S1.** The RMSE in different models.**Table S2.** World population age standard. **Table S3.** The age-standardized rate of breast cancer case in the world, East Asia and Pacific, China, Japan and South Korea from 1990 to 2019. **Table S4.** EAPC of percent of DALYs and deaths for breast cancer attributable to risk factors in China, Japan and South Korea, 1990-2019. **Table S5.** Predicted number of breast cancer incident cases and deaths in China, Japan and South Korea, and changes between 2020 and 2034 stratified into changes due to risk and demographics by sex.  **Table S6.** Number of population estimates (in millions) in 1990 and 2019 and population projections in 2020 and 2034 by country.  **Table S7.** The death rates of breast cancer in China, Japan and South Korea by sex and age, during 1990-2019, and projected until 2034.

## Data Availability

The data that support the findings of this study are openly available in Global Health Data Exchange, at http://ghdx.healthdata.org/gbd-results-tool. Further information is available from the corresponding author upon request.

## Abbreviations

APC: Age-period-cohort
ASDR: Age-standardized death rate
ASIR: Age-standardized incidence rate
ASR: Age-standardized rate
BAPC: Bayesian age-period-cohort
BAMP: Bayesian age-period-cohort modeling and prediction
BC: Breast cancer
BMI: Body mass index
CI: Confidence interval
CI5: Cancer Incidence in Five Continents
CRA: Comparative risk assessment
DALYs: Disability-adjusted life-years
EAPC: Estimated annual percentage change
ER: Exponential regression
FPG: Fasting plasma glucose
GBD: Global Burden of Diseases
HER2: Human epidermal growth factor receptor 2
RMSE: Root-mean-squared error
RW: Random walk
SDI: Socio-demographic index
UI: Uncertainty interval
