# Supplementary material for: Burden, trends, and risk factors for breast cancer in China from 1990 to 2019 and its predictions until 2034: an up-to-date overview and comparison with those in Japan and South Korea
Source: BMC Cancer. 2022 Jul 29;22:826. doi: 10.1186/s12885-022-09923-4 (PMC9334732; doi:10.1186/s12885-022-09923-4)
Supplement: Supplementary file 1 — Additional file 1: Fig S1. The number of breast cancer incident cases, deaths and disability-adjusted life years (DALYs) by sex and location from1990 to 2019. (A-C) Both sexes; (D-F) Female; (G-I) Male. Fig S2. The ASIR, ASDR, and ASR of DALYs of breast cancer in China, Japan, South Korea,East Asia and Pacific and the world from 1990 to 2019. (A-C) Both sexes; (D-F) Female; (G-I) Male. ASIR: age-standardized incidence rate, ASDR: age-standardized death rate, ASR:age-standardized rate, DALYs: disability-adjusted life-years. Fig S3. The number of breast cancer incident case, deaths and disability-adjusted life years (DALYs) for both sexes by age group in China, Japan, South Korea, East Asia and the Pacific and the world in 2019. The error bar indicates 95% uncertainty interval of the number. Fig S4. The rates of breast cancer incidence, death and disability-adjusted life years (DALYs) for both sexes by age group in China, Japan, South Korea, East Asia and the Pacific and the world in 2019. The error bar indicates 95% uncertainty interval of the rates. Fig S5. The EAPCs of ASIR, ASDR and ASR of DALYs for breast cancer from 1990 to 2019 by sex in the China, Japan, South Korea, East Asia and the Pacific and the world. EAPC: estimated annual percentage change; ASIR: age-standardized incidence rate; ASDR: age-standardized death rate; ASR: age-standardized rate; DALYs: disability-adjusted life-years. The error bar indicates 95% confidence interval (CI) of the EAPCs. Fig S6. Temporal trends of percent of deaths for breast cancer attributable to risk factors in China(A), Japan (B) and South Korea (C), 1990-2019. Fig S7. Temporal trends of percent of DALYs for breast cancer attributable to risk factors in China(A), Japan (B) and South Korea (C), 1990-2019. DALYs: disability-adjusted life-years. Fig S8. Trends in ASIR and ASDR during 1990–2019 and predictions from 2020 to 2034 in China(A-B), Japan (C-D) and South Korea (E-F). ASIR: age-standardized incidence rate; ASDR: age-sta [file 12885_2022_9923_MOESM1_ESM.pdf]

Table of Contents

Table S1. The RMSE in different models.

Table S2. World population age standard

Table S3. The age-standardized rate of breast cancer case in the world, East Asia and Pacific, China, Japan and South Korea from 1990 to 2019

Table S4. EAPC of percent of DALYs and deaths for breast cancer attributable to risk factors in China, Japan and South Korea, 1990-2019

Table S5. Predicted number of breast cancer incident cases and deaths in China, Japan and South Korea, and changes between 2020 and 2034 stratified into changes due to risk and demographics by sex.

Table S6. Number of population estimates (in millions) in 1990 and 2019 and population projections in 2020 and 2034 by country.

Table S7. The death rates of breast cancer in China, Japan and South Korea by sex and age, during 1990-2019, and projected until 2034.

Fig S1. The number of breast cancer incident cases (A), deaths (B) and disability-adjusted life years (DALYs) (C) by sex and location from1990 to 2019

Fig S2. The ASIR(A), ASDR(B), and ASR of DALYs(C) of breast cancer in China, Japan, South Korea, East Asia and Pacific and the world from 1990 to 2019. ASIR: age-standardized incidence rate, ASDR: age-standardized death rate, ASR: age-standardized rate, DALYs: disability-adjusted life-years

Fig S3. The number of breast cancer incident case, deaths and disability-adjusted life years (DALYs) for both sexes by age group in China, Japan, South Korea, East Asia and the Pacific and the world in 2019. The error bar indicates 95% uncertainty interval of the number

Fig S4. The rates of breast cancer incidence, death and disability-adjusted life years (DALYs) for both sexes by age group in China, Japan, South Korea, East Asia and the Pacific and the world in 2019. The error bar indicates 95% uncertainty interval of the rates

Fig S5. The EAPCs of ASIR, ASDR and ASR of DALYs for breast cancer from 1990 to 2019 by

sex in the China, Japan, South Korea, East Asia and the Pacific and the world. EAPC: estimated annual percentage change; ASIR: age-standardized incidence rate; ASDR: age-standardized death rate; ASR: age-standardized rate; DALYs: disability-adjusted life-years. The error bar indicates 95% confidence interval (CI) of the EAPCs

Fig S6. Temporal trends of percent of deaths for breast cancer attributable to risk factors in China (A), Japan (B) and South Korea (C), 1990-2019.

Fig S7. Temporal trends of percent of DALYs for breast cancer attributable to risk factors in China (A), Japan (B) and South Korea (C), 1990-2019. DALYs: disability-adjusted life-years

Fig S8. Trends in ASIR and ASDR during 1990–2019 and predictions from 2020 to 2034 in China (A-B), Japan (C-D) and South Korea (E-F). ASIR: age-standardized incidence rate; ASDR: age-standardized death rate

Fig S9. The number of incident cases and deaths during 1990-2019 and projected from 2020 to 2034 in Japan. (A-B) Both sexes; (C-D) Female; (E-F) Male. The error bar indicates 95% confidence interval (CI) of the number

Fig S10. The number of incident cases and deaths during 1990-2019 and projected from 2020 to 2034 in South Korea. (A-B) Both sexes; (C-D) Female; (E-F) Male. The error bar indicates 95% confidence interval (CI) of the number

Table S1. The RMSE in different models.

Models	incidence			death		
	China	Japan	South Korea	China	Japan	South Korea
BAPC	48.8063	16.0654	1.4269	**1.7219***	1.5266	0.1873
ER	19.3235	12.4473	4.2027	5.7156	1.0471	0.3427
BAMP(RW1)	25.2149	**1.8185***	**0.7931***	2.0723	**0.5573***	**0.0487***
BAMP(RW2)	**7.6281***	15.3948	1.0358	2.7782	1.823	0.1797
CR	61.2986	38.3248	2.6009	10.3053	1.0458	0.4293

* denotes the lowest RMSE.

RMSE, root-mean-squared-error, reflects the average deviation of estimates from the observed counts during the validation period

Table S2. World population age standard

Age group	Percent of Population
0-4	9.94
5-9	9.57
10-14	8.99
15-19	8.32
20-24	7.87
25-29	7.63
30-34	7.33
35-39	6.81
40-44	6.14
45-49	5.51
50-54	4.92
55-59	4.35
60-64	3.68
65-69	2.99
70-74	2.27
75-79	1.61
80-84	1.11
85+	0.96

Table S3. The age-standardized rate of breast cancer case in the world, East Asia and Pacific, China, Japan and South Korea from 1990 to 2019

Variables	ASIR(/10^5)/(95%UI)		EAPC (95%CI)	ASDR(/10^5)/(95%UI)		EAPC (95%CI)	ASR of DALYs (/10^5) (95%CI)		EAPC (95%CI)
	1990	2019	ASIR (1990-2019)	1990	2019	ASDR (1990-2019)	1990	2019	ASR of DALYRs (1990-2019)
World									
Both	21.44(20.65-22.1)	24.17(22.11-26.24)	0.32(0.27-0.38) *	9.8(9.3-10.21)	8.62(7.95-9.25)	-0.56(-0.61--0.51) *	275.32(263.24-288.77)	247.63(228.68-266.08)	-0.51(-0.56--0.45) *
Female	40.12(38.78-41.33)	45.86(41.91-49.76)	0.36(0.31-0.42) *	17.76(16.93-18.51)	15.88(14.66-17.07)	-0.51(-0.57--0.46) *	524.87(501.78-551.15)	473.83(437.3-510.51)	-0.51(-0.57--0.45) *
Male	0.53(0.5-0.57)	0.65(0.58-0.72)	0.9(0.75-1.06) *	0.37(0.34-0.40)	0.33(0.29-0.36)	-0.23(-0.37--0.09)	8.18(7.54-8.82)	7.96(7.03-8.8)	0.14(-0.01-0.29)
East Asia and Pacific									
Both	11.73(10.69-12.84)	20.5(17.64-23.7)	2.03(1.95-2.12) *	6.06(5.5-6.67)	6.24(5.52-7.02)	0.08(0.05-0.11)	190.18(171.85-210.91)	193.3(171.67-217.67)	-0.01(-0.05-0.04)
Female	22.83(20.74-25.05)	39.61(34-45.99)	1.98(1.9-2.06) *	11.49(10.42-12.64)	11.67(10.32-13.2)	0(-0.04-0.05)	375.26(338.26-417.02)	374.63(331.57-423.38)	-0.1(-0.16--0.04)
Male	0.21(0.19-0.24)	0.59(0.48-0.71)	4.99(4.31-5.68) *	0.17(0.14-0.19)	0.26(0.22-0.31)	2.78(2.22-3.33) *	3.66(3.19-4.15)	6.89(5.68-8.25)	3.62(2.98-4.26) *
China									
Both	8.54(7.07-10.11)	18.32(14.5-22.93)	2.8(2.7-2.91) *	4.74(3.96-5.57)	4.85(3.91-5.92)	0.06(0-0.12)	145.67(121.08-172.57)	144.15(117.26-174.99)	-0.13(-0.19--0.06) *
Female	17.07(14.02-20.3)	35.61(28.07-44.81)	2.66(2.57-2.76) *	9.16(7.61-10.82)	9.02(7.19-11.1)	-0.14(-0.21--0.06) *	294.04(243.51-349.12)	277.98(224.35-339.93)	-0.35(-0.43--0.26) *
Male	0.13(0.1-0.15)	0.69(0.53-0.88)	8.37(7.32-9.43) *	0.1(0.08-0.12)	0.29(0.22-0.36)	6.03(5.08-6.97) *	2.45(2-2.93)	7.73(5.98-9.62)	6.38(5.37-7.39) *
Japan									
Both	19.07(17.92-20.29)	31.02(24.85-38.16)	1.94(1.71-2.18) *	4.82(4.59-4.95)	5.37(4.85-5.72)	0.55(0.41-0.7) *	157.67(151.39-164.52)	172.96(160.51-187.61)	0.42(0.24-0.6) *
Female	36.07(33.92-38.39)	60.47(48.35-74.56)	2.03(1.8-2.27) *	8.8(8.42-9.03)	10.14(9.24-10.78)	0.66(0.5-0.81) *	300.26(288.72-313.02)	338.09(314.53-366.48)	0.5(0.31-0.68) *
Male	0.19(0.17-0.22)	0.24(0.19-0.31)	0.65(0.36-0.93) *	0.09(0.08-0.09)	0.08(0.07-0.09)	-0.7(-0.96--0.44) *	2.06(1.98-2.15)	1.91(1.77-2.07)	-0.59(-0.84--0.34)
South Korea									
Both	9.5(8.9-10.19)	23.79(19.29-28.65)	3.42(3.07-3.78) *	4.32(4.09-4.58)	4.69(4.12-5.24)	0.46(0.31-0.6) *	136.52(129.1-144.82)	143.56(126.99-161.59)	0.39(0.26-0.52) *
Female	17.5(16.39-18.81)	46.4(37.59-56.14)	3.6(3.27-3.94) *	7.62(7.21-8.08)	8.73(7.71-9.73)	0.64(0.52-0.76) *	255.11(241.07-270.67)	281.19(247.83-316.56)	0.53(0.42-0.64) *
Male	0.13(0.11-0.15)	0.21(0.15-0.28)	0.52(-0.04-1.09)	0.09(0.08-0.11)	0.08(0.06-0.1)	-1.89(-2.53--1.26) *	2.03(1.75-2.4)	1.77(1.4-2.2)	-1.61(-2.13--1.09)

ASIR: age-standardized incidence rate, ASDR: age-standardized death rate, ASR: age-standardized rate, DALYs: disability-adjusted life-years, EAPC: estimated annual percentage change

* Changes that are statistically significant.

Table S4. EAPC of percent of DALYs and deaths for breast cancer attributable to risk factors in China, Japan and South Korea, 1990-2019

Country	Sex	Risk factors	Measure	EAPC (95%CI)
China	Female	Alcohol use	DALYs_percent	0.5(0.25-0.75) *
China	Female	Diet high in red meat	DALYs_percent	1.65(1.58-1.72) *
China	Female	High body-mass index	DALYs_percent	3.22(3.03-3.41) *
China	Female	High fasting plasma glucose	DALYs_percent	0.85(0.62-1.08) *
China	Female	Low physical activity	DALYs_percent	-0.14(-0.33-0.04)
China	Female	Tobacco	DALYs_percent	-0.24(-0.28--0.19) *
China	Male	Alcohol use	DALYs_percent	0.46(0.33-0.6) *
China	Male	Diet high in red meat	DALYs_percent	1.9(1.79-2.01) *
China	Male	Tobacco	DALYs_percent	-0.19(-0.22--0.16) *
Japan	Female	Alcohol use	DALYs_percent	-0.49(-0.55--0.42) *
Japan	Female	Diet high in red meat	DALYs_percent	-0.15(-0.24--0.05)
Japan	Female	High body-mass index	DALYs_percent	4.23(3.92-4.54) *
Japan	Female	High fasting plasma glucose	DALYs_percent	0.32(0.09-0.55) *
Japan	Female	Low physical activity	DALYs_percent	0.43(0.36-0.49) *
Japan	Female	Tobacco	DALYs_percent	-0.36(-0.41--0.31) *
Japan	Male	Alcohol use	DALYs_percent	-0.3(-0.35--0.26) *
Japan	Male	Diet high in red meat	DALYs_percent	-0.09(-0.18-0.01) *
Japan	Male	Tobacco	DALYs_percent	-0.39(-0.45--0.34) *
South Korea	Female	Alcohol use	DALYs_percent	0.06(-0.01-0.13)
South Korea	Female	Diet high in red meat	DALYs_percent	1.82(1.4-2.23) *
South Korea	Female	High body-mass index	DALYs_percent	4.28(3.75-4.81) *
South Korea	Female	High fasting plasma glucose	DALYs_percent	1.09(0.94-1.25) *
South Korea	Female	Low physical activity	DALYs_percent	0.5(0.47-0.54) *
South Korea	Female	Tobacco	DALYs_percent	-1.42(-1.48--1.35) *
South Korea	Male	Alcohol use	DALYs_percent	0.12(0.07-0.17)
South Korea	Male	Diet high in red meat	DALYs_percent	1.81(1.44-2.18) *
South Korea	Male	Tobacco	DALYs_percent	-0.35(-0.37--0.33) *
China	Female	Alcohol use	Death_percent	0.35(0.18-0.52) *
China	Female	Diet high in red meat	Death_percent	1.6(1.53-1.67) *
China	Female	High body-mass index	Death_percent	2.99(2.86-3.12) *
China	Female	High fasting plasma glucose	Death_percent	0.72(0.5-0.95) *
China	Female	Low physical activity	Death_percent	-0.25(-0.41--0.09) *
China	Female	Tobacco	Death_percent	-0.26(-0.33--0.19) *
China	Male	Alcohol use	Death_percent	0.46(0.32-0.61) *
China	Male	Diet high in red meat	Death_percent	1.77(1.67-1.87) *
China	Male	Tobacco	Death_percent	-0.37(-0.42--0.32) *
Japan	Female	Alcohol use	Death_percent	-0.54(-0.62--0.46) *
Japan	Female	Diet high in red meat	Death_percent	-0.16(-0.25--0.06) *
Japan	Female	High body-mass index	Death_percent	1.64(1.52-1.75) *
Japan	Female	High fasting plasma glucose	Death_percent	0.15(-0.09-0.39) *

Japan	Female	Low physical activity	Death_percent	0.41(0.32-0.49) *
Japan	Female	Tobacco	Death_percent	-0.55(-0.6--0.51) *
Japan	Male	Alcohol use	Death_percent	-0.34(-0.39--0.29) *
Japan	Male	Diet high in red meat	Death_percent	-0.08(-0.17-0) *
Japan	Male	Tobacco	Death_percent	-0.39(-0.45--0.34) *
South Korea	Female	Alcohol use	Death_percent	0.04(-0.04-0.12)
South Korea	Female	Diet high in red meat	Death_percent	1.66(1.26-2.07) *
South Korea	Female	High body-mass index	Death_percent	2.04(1.86-2.22) *
South Korea	Female	High fasting plasma glucose	Death_percent	1.06(0.89-1.22) *
South Korea	Female	Low physical activity	Death_percent	0.53(0.49-0.58) *
South Korea	Female	Tobacco	Death_percent	-1.49(-1.55--1.43) *
South Korea	Male	Alcohol use	Death_percent	0.19(0.12-0.25)
South Korea	Male	Diet high in red meat	Death_percent	1.69(1.35-2.03) *
South Korea	Male	Tobacco	Death_percent	-0.27(-0.3--0.24) *

DALYs: disability-adjusted life years

* Changes that are statistically significant.

Table S5. Predicted number of breast cancer incident cases and deaths in China, Japan and South Korea, and changes between 2020 and 2034 stratified into changes due to risk and demographics by sex.

Country	Number of incident cases or deaths				Changes between 2020 and 2034 (%)			
	2020	2025	2030	2034	Total change	Change due to risk	Change due to age structure	Change due to population
China								
incidence								
female	361,917	393,089	419,673	434,744	20.5	0.7	17.39	2.41
male	7,621	9,583	11,497	13,105	72.52	32.56	38.59	1.37
death								
female	89,648	98,531	105,954	110,777	23.09	-11.62	32.27	2.44
male	2,887	3,674	4,415	5,055	73.82	27.63	44.81	1.38
Japan								
incidence								
female	73,786	74,806	74,602	73,555	-0.33	0.36	6.03	-6.72
male	329	343	354	362	10.21	0.11	17.81	-7.71
death								
female	15,951	16,760	17,393	17,585	10.51	0.93	16.65	-7.07
male	120	128	134	139	15.13	-5.97	29.01	-7.91
South Korea								
incidence								
female	20,046	21,689	23,001	23,622	17.57	-2.28	20.43	-0.58
male	68	81	94	103	55.56	0.35	56.82	-1.61
death								
female	4,076	4,539	4,927	5,181	27.17	-6.79	34.57	-0.61
male	21	27	32	36	75	1.22	75.52	-1.74

Table S6. Number of population estimates (in millions) in 1990 and 2019 and population projections in 2020 and 2034 by country.

Variable	Value	China				Japan				South Korea			
		1990	2019	2020	2034	1990	2019	2020	2034	1990	2019	2020	2034
Female	Number	573.5	697.53	701.08	716.59	63.99	65.52	64.72	60.51	22.07	26.42	25.6	25.47
15-49	Number	323.02	351.1	342.28	306.07	32.13	25.41	24.84	20.17	12.64	12.37	11.74	9.25
	Percent	56.33%	50.33%	48.82%	42.71%	50.20%	38.79%	38.37%	33.33%	57.30%	46.83%	45.85%	36.34%
50-69	Number	74.26	184.39	186.1	204.8	14.58	16.58	16.26	16.88	3.1	7.62	7.54	7.92
	Percent	12.95%	26.43%	26.54%	28.58%	22.78%	25.31%	25.12%	27.90%	14.03%	28.84%	29.43%	31.11%
>70	Number	21.75	58.72	53.89	101.28	6.05	15.92	15.97	17.16	0.83	3.1	3.21	5.76
	Percent	3.79%	8.42%	7.69%	14.13%	9.45%	24.30%	24.67%	28.36%	3.77%	11.74%	12.53%	22.63%
Male	Number	610.19	724.82	738.25	745.88	61.88	62.27	61.75	57.4	22.27	26.98	25.67	25.34
15-49	Number	345.44	369.63	370.15	342.17	32.83	26.34	25.94	21.13	13.24	13.68	12.75	10.2
	Percent	56.61%	51%	50.14%	45.87%	53.06%	42.30%	42.01%	36.82%	59.44%	50.69%	49.69%	40.25%
50-69	Number	79.79	184.5	187.75	206.74	13.38	16.28	16.16	16.93	2.65	7.62	7.39	7.97
	Percent	13.08%	25.45%	25.43%	27.72%	21.62%	26.14%	26.17%	29.50%	11.92%	28.25%	28.81%	31.44%
>70	Number	16.51	49.24	44.22	83	3.84	11.65	11.57	12.69	0.44	2.15	2.21	4.5
	Percent	2.71%	6.79%	5.99%	11.13%	6.20%	18.70%	18.73%	22.10%	1.97%	7.97%	8.61%	17.76%

Table S7. The death rates of breast cancer in China, Japan and South Korea by sex and age, during 1990-2019, and projected until 2034.

	Death Rate (/10^5)				EAPC	
	1990 (95%UI)	2019 (95%UI)	2020 (95%CI)	2034 (95%CI)	1990-2019 (95%CI)	2020-2034 (95%CI)
China						
Both [a]	4.74(3.96-5.57)	4.85(3.91-5.92)	4.76(4.06-5.57)	4.41(3.6-5.43)	0.06(0-0.12) *	-0.55(-0.58--0.52) *
Female [a]	9.16(7.61-10.82)	9.02(7.19-11.1)	8.85(7.51-10.44)	8(6.48-9.92)	-0.14(-0.21--0.06) *	-0.74(-0.78--0.7) *
All ages	7.22(5.96-8.57)	13.4(10.68-16.55)	12.79(11.93-13.72)	15.46(13.39-18.01)	2.09(2.02-2.16) *	1.34(1.27-1.4) *
15-49	3.9(3.12-4.75)	4.53(3.52-5.78)	4.57(3.86-5.41)	4.11(3.29-5.16)	-0.08(-0.48-0.33)	-0.006(-0.008--0.004) *
50-69	28.07(23.07-33.41)	26.27(20.74-32.83)	25.12(21.42-29.670	22.71（18.43-28.03)	-0.13(-0.31-0.04)	-0.64(-0.74--0.54) *
70+	36.77(31.66-42.91)	49.66(40.36-59.81)	50.03(42.47-58.94)	50.45(40.83-62.05)	1.11(1.04-1.19) *	0.05(0-0.1)
Male [a]	0.1(0.08-0.12)	0.29(0.22-0.36)	0.29(0.23-0.36)	0.36(0.18-0.71)	6.03(5.08-6.97) *	1.55(1.45-1.65) *
All ages	0.06(0.05-0.07)	0.39(0.3-0.49)	0.39(0.32-0.47)	0.68(0.35-1.32)	8.98(7.93-10.03) *	3.9(3.77-4.03) *
15-49	0.02(0.02-0.02)	0.08(0.06-0.1)	0.07(0.05-0.1)	0.05(0.02-0.11)	7.27(6.16-8.38) *	-1.86(-2.14--1.58) *
50-69	0.25(0.2-0.32)	0.88(0.65-1.14)	0.95(0.75-1.21)	1.33(0.67-2.63)	7.02(5.89-8.15) *	2.65(2.47-2.84) *
70+	0.64(0.52-0.76)	1.8(1.43-2.220	1.87(1.45-2.4)	2.54(1.27-5)	5.33(4.59-6.08) *	1.9(1.68-2.11) *
Japan						
Both [a]	4.82(4.59-4.95)	5.37(4.85-5.72)	5.41(4.97-5.88)	5.01(4.32-5.81)	0.55(0.41-0.7) *	-0.54(-0.56--0.52) *
Female [a]	8.8(8.42-9.03)	10.14(9.24-10.78)	10.2(9.4-11.08)	9.35(8.07-10.85)	0.66(0.5-0.81) *	-0.62(-0.64--0.6) *
All ages	12.33(11.79-12.65)	24.29(20.41-26.39)	24.64(23.53-25.72)	29.06(25.7-32.96)	2.53(2.39-2.68) *	1.21(1.15-1.26) *
15-49	6.02(5.88-6.16)	5.99(5.73-6.34)	5.74(4.96-6.58)	4.67(3.69-5.78)	-0.24(-0.38--0.1) *	-1.5(-1.69--1.31) *
50-69	25.28(24.51-25.91)	32.75(30.92-34.55)	32.59(29.57-35.88)	26.66(22.88-30.99)	1.14(0.93-1.36) *	-1.41(-1.49--1.34) *
70+	37.52(33.46-39.42)	56.27(42.17-63.79)	57.71(52.75-63.06)	70.79(61.31-81.92)	1.87(1.68-2.05) *	1.5(1.46-1.57) *
Male [a]	0.09(0.08-0.09)	0.08(0.07-0.09)	0.07(0.06-0.09)	0.07(0.05-0.1)	-0.7(-0.96--0.44) *	-0.25(-0.27--0.24) *
All ages	0.1(0.09-0.1)	0.2(0.18-0.22)	0.19(0.15-0.24)	0.24(0.18-0.33)	2.22(1.93-2.51) *	1.56(1.5-1.62) *
15-49	0.02(0.02-0.02)	002(0.02-0.02)	0.01(0.01-0.02)	0.005(0-0.02)	0	-4(-6.78--1.22) *
50-69	0.18(0.17-0.19)	0.18(0.17-0.2)	0.17(0.06-0.32)	0.17(0.06-0.32)	-0.28(-0.56--0.01) *	-0.1(-0.54--0.35) *

70+	0.77(0.72-0.81)	0.8(0.68-0.88)	0.77(0.44-1.15)	0.85(0.47-1.31)	-0.19(-0.4-0.02)	0.77(0.65-0.89) *
South Korea						
Both [a]	4.32(4.09-4.58)	4.69(4.12-5.24)	4.67(4.23-5.17)	4.32(3.65-5.08)	0.46(0.31-0.6) *	-0.57(-0.6--0.53) *
Female [a]	7.62(7.21-8.08)	8.73(7.71-9.73)	8.66(7.82-9.6)	7.97(6.72-9.4)	0.64(0.52-0.76) *	-0.61(-0.65--0.57) *
All ages	6.43(6.09-6.81)	15.38(13.56-17.22)	15.92(15.05-16.88)	20.34(17.78-23.21)	3.24(3.13-3.36) *	1.75(1.66-1.83) *
15-49	3.8(3.56-4.09)	5.03(4.29-5.81)	4.86(3.94-5.92)	4.14(3.01-5.46)	1.23(0.98-1.49) *	-1.21(-1.31--1.1) *
50-69	22.52(20.89-24.46)	25.07(21.61-28.62)	24.63(21.48-28.08)	23.12(19.15-27.72)	0.43(0.34-0.53) *	-0.45(-0.57--0.33) *
70+	29.08(26.43-32.15)	49.33(40.36-57.24)	51.23(44.43-58.59)	51.38(43.12-60.8)	2.03(1.85-2.21) *	-0.06(-0.15-0.03)
Male [a]	0.09(0.08-0.11)	0.08(0.06-0.1)	0.06(0.04-0.09)	0.05(0.03-0.09)	-1.89(-2.53--1.26) *	-0.29(-0.31--0.27) *
All ages	0.04(0.04-0.05)	0.1(0.08-0.13)	0.09(0.05-0.13)	0.14(0.08-0.22)	1.98(1.51-2.44) *	3.84(3.57-4.12) *
15-49	0.02(0.01-0.02)	0.02(0.01-0.02)	NA	NA	0	NA
50-69	0.16(0.12-0.21)	0.14(0.09-0.21)	0.12(0-0.32)	0.14(0.01-0.34)	-1.61(-2.16--1.06) *	0.66(0.21-1.1) *
70+	0.65(0.48-0.87)	0.68(0.47-0.97)	0.5(0-1.36)	0.53(0.11-1.20	-1.39(-2.08--0.7) *	0.11(-0.22-0.44) *

EAPC: estimated annual percentage change; [a], Age-standardized death rate; [*] Changes that are statistically significant.

Figure S1

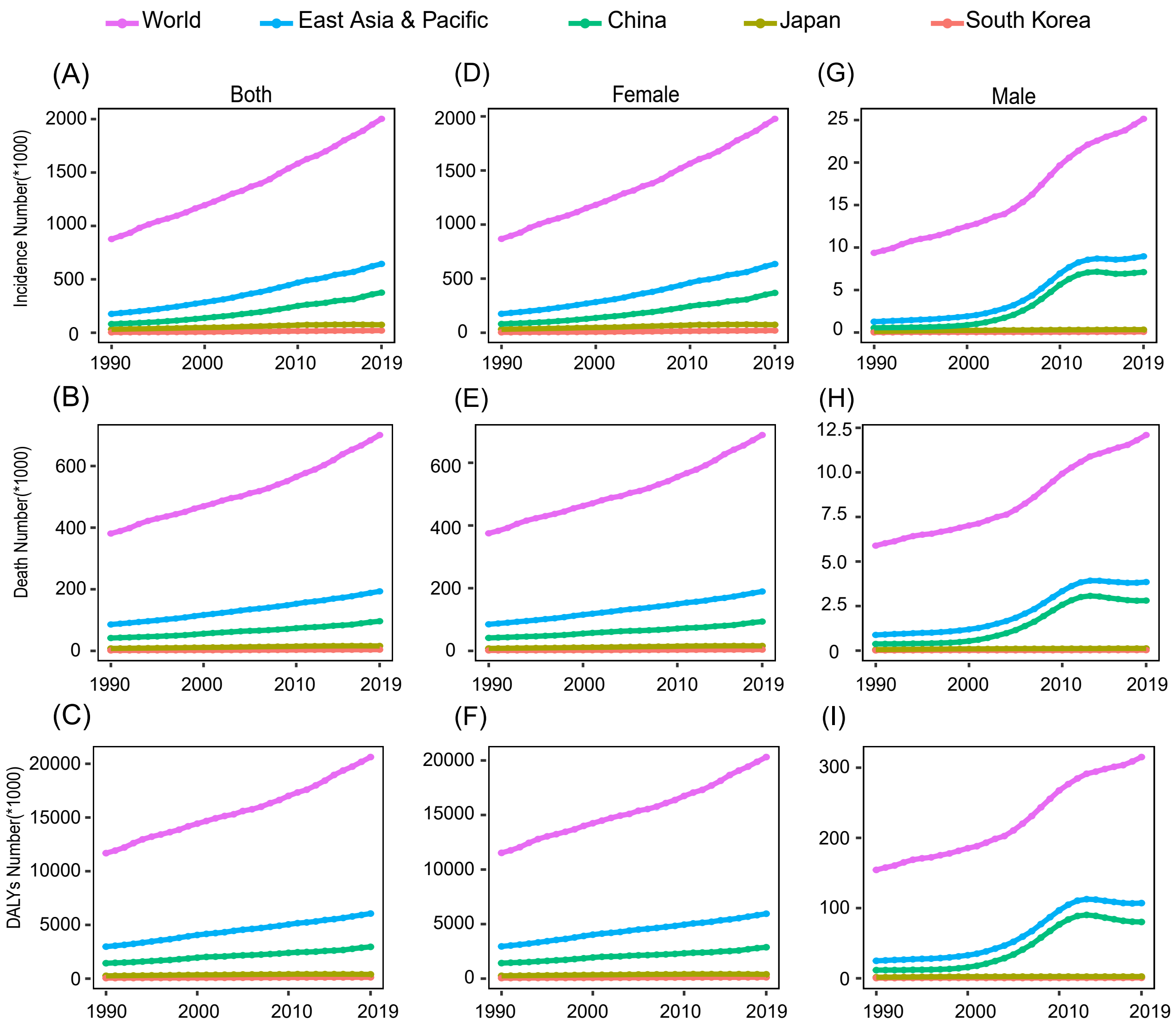

World
East Asia & Pacific
China
Japan
South Korea
(A)
Both
(D)
Female
(G)
Male
Incidence Number(*1000)
2000
1500
1000
500
0
25
20
15
10
5
1990
2000
2010
2019
(B)
(E)
(H)
Death Number(*1000)
600
400
200
12.5
10.0
7.5
5.0
2.5
(C)
(F)
(I)
DALYs Number(*1000)
20000
15000
10000
5000
300
200
100

Figure S2

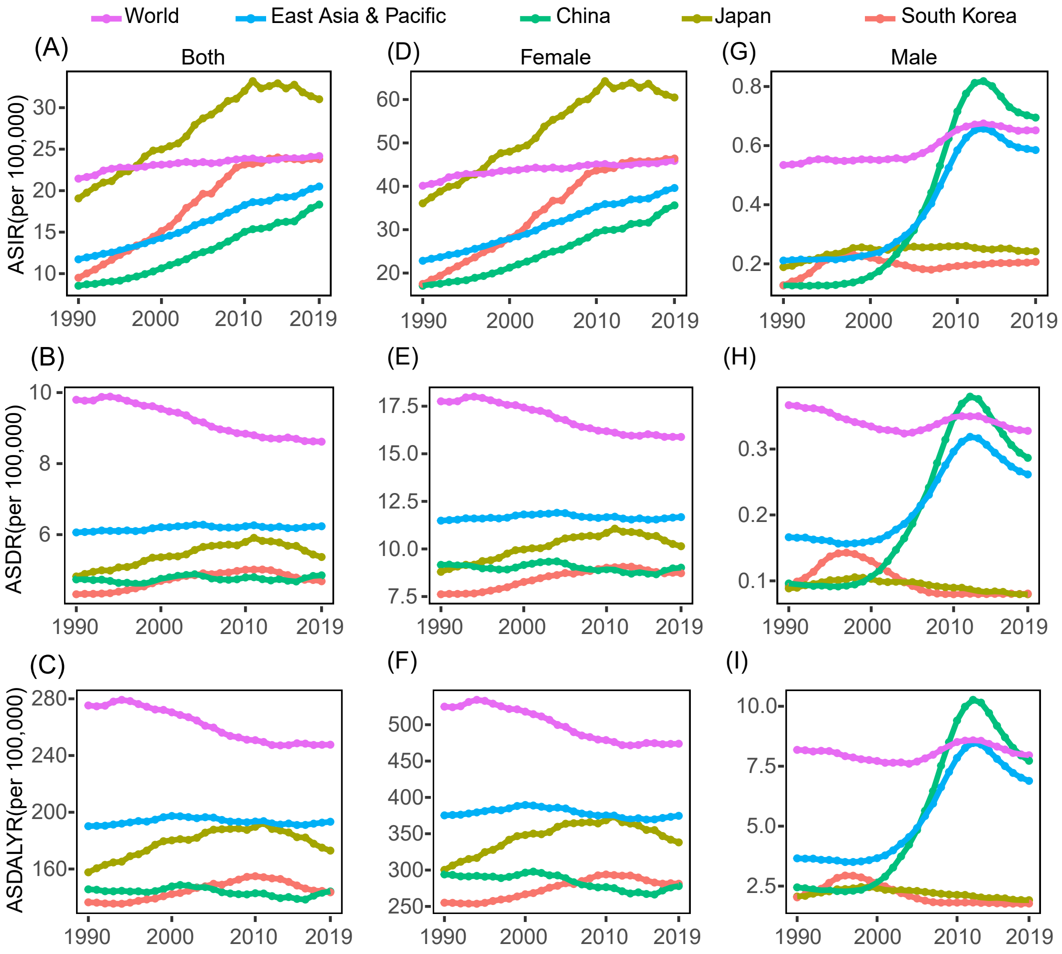
World
East Asia & Pacific
China
Japan
South Korea
(A)
Both
(D)
Female
(G)
Male
ASIR(per 100,000)
(B)
(E)
(H)
ASDR(per 100,000)
(C)
(F)
(I)
ASDALYR(per 100,000)
1990
2000
2010
2019

Figure S3

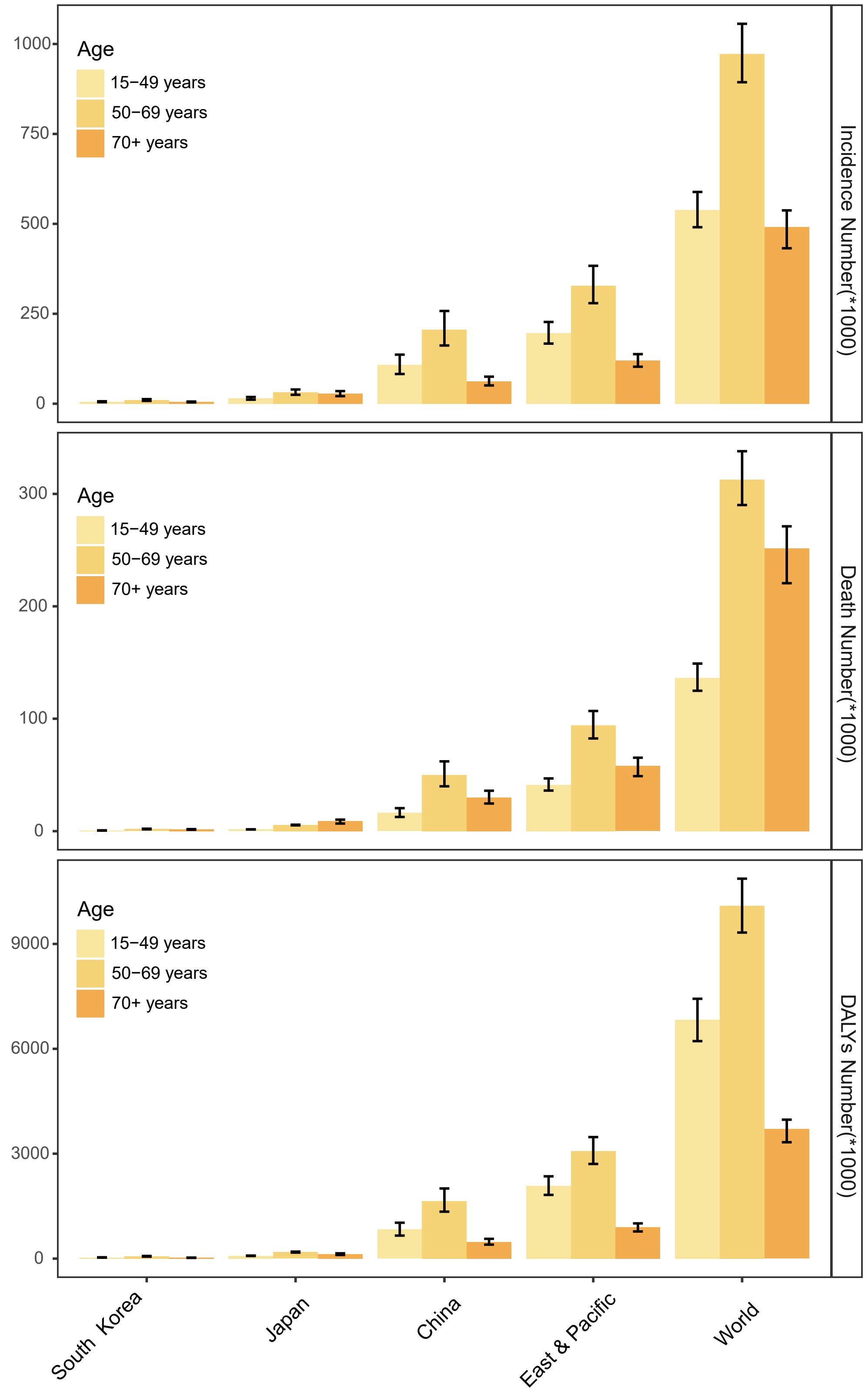

Age
15−49 years
50−69 years
70+ years
0
250
500
750
1000
Incidence Number(*1000)
Age
15−49 years
50−69 years
70+ years
0
100
200
300
Death Number(*1000)
Age
15−49 years
50−69 years
70+ years
0
3000
6000
9000
DALYs Number(*1000)
South Korea
Japan
China
East & Pacific
World

Figure S4

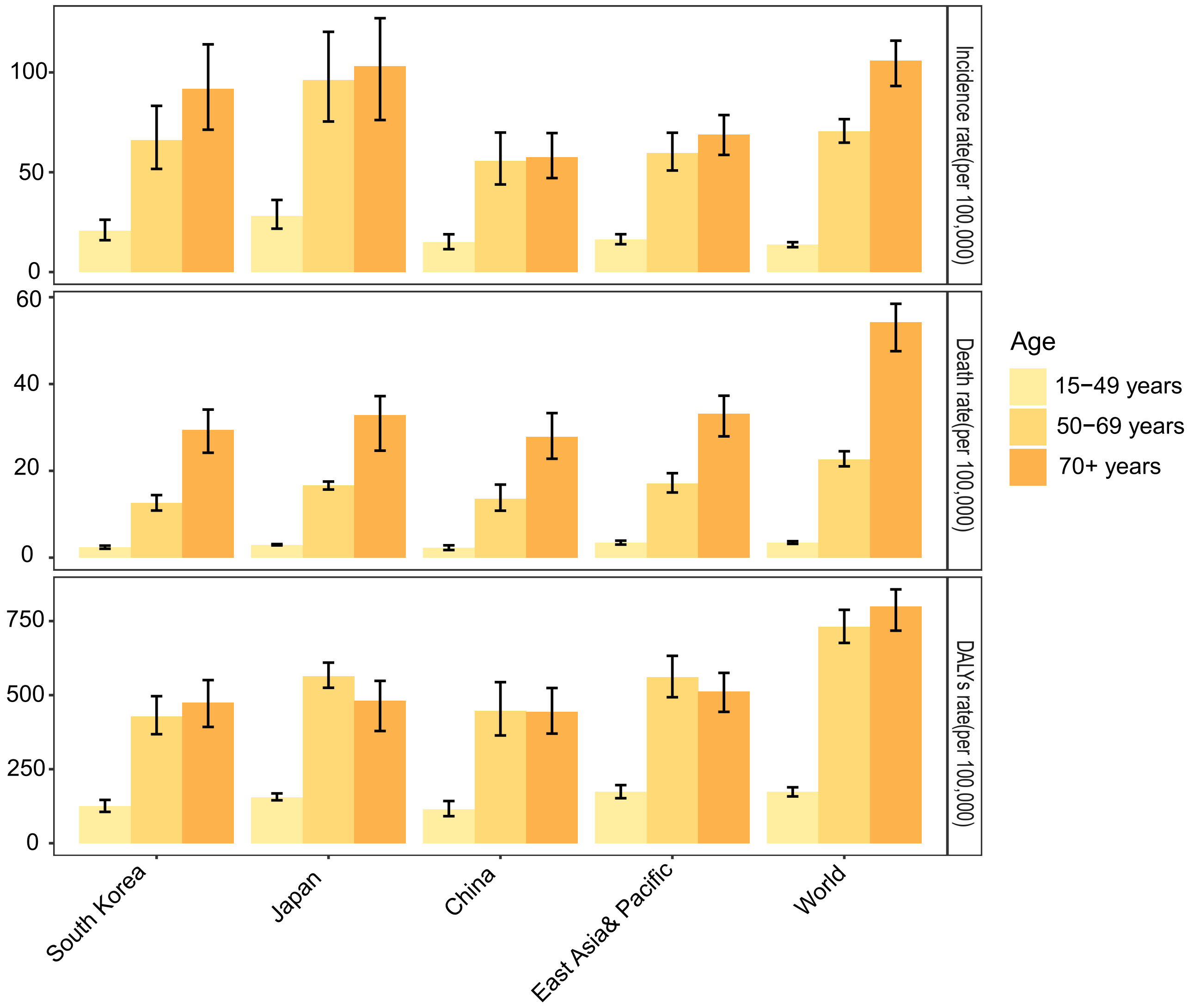

Incidence rate(per 100,000)
Death rate(per 100,000)
DALYs rate(per 100,000)
100
50
0
60
40
20
0
750
500
250
0
Age
15−49 years
50−69 years
70+ years
South Korea
Japan
China
East Asia& Pacific
World

Figure S5

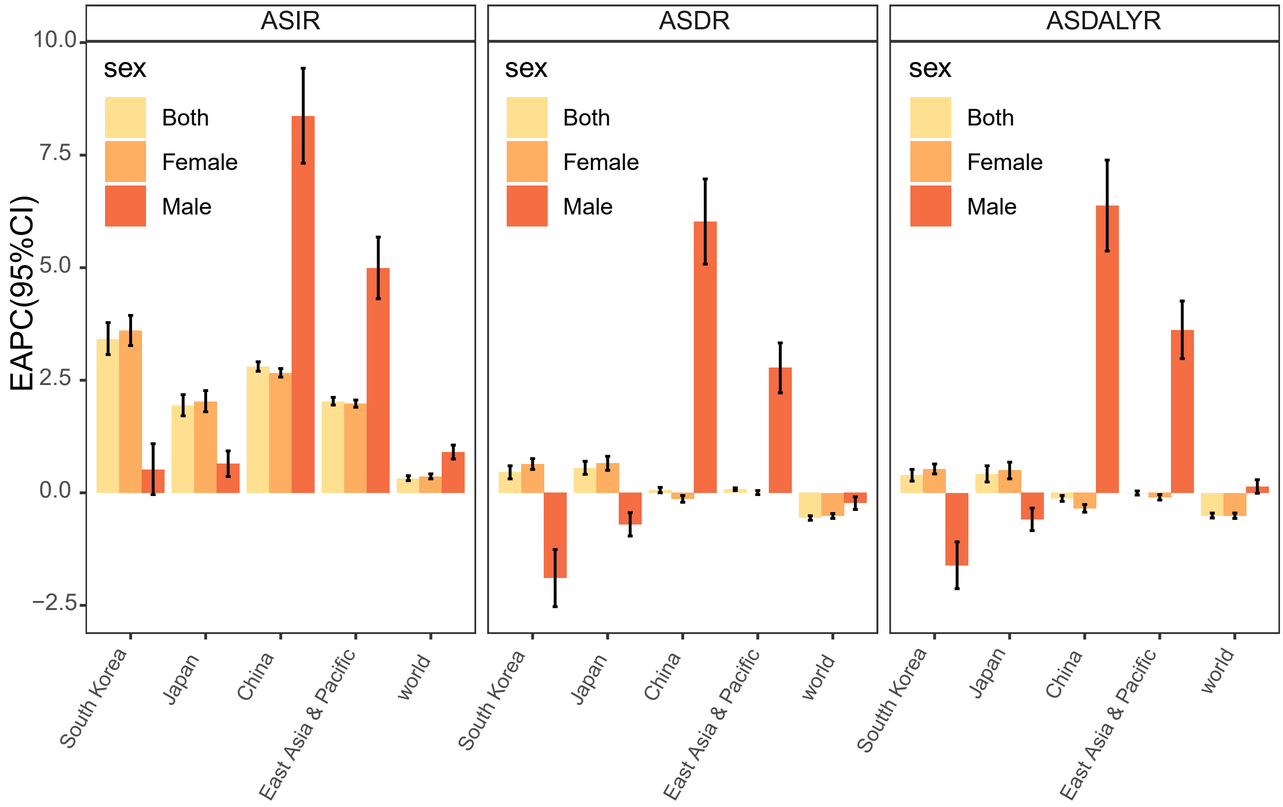

ASIR
ASDR
ASDALYR
sex
Both
Female
Male
EAPC(95%CI)
10.0
7.5
5.0
2.5
0.0
−2.5
South Korea
Japan
China
East Asia & Pacific
world
Soutth Korea

Figure S6

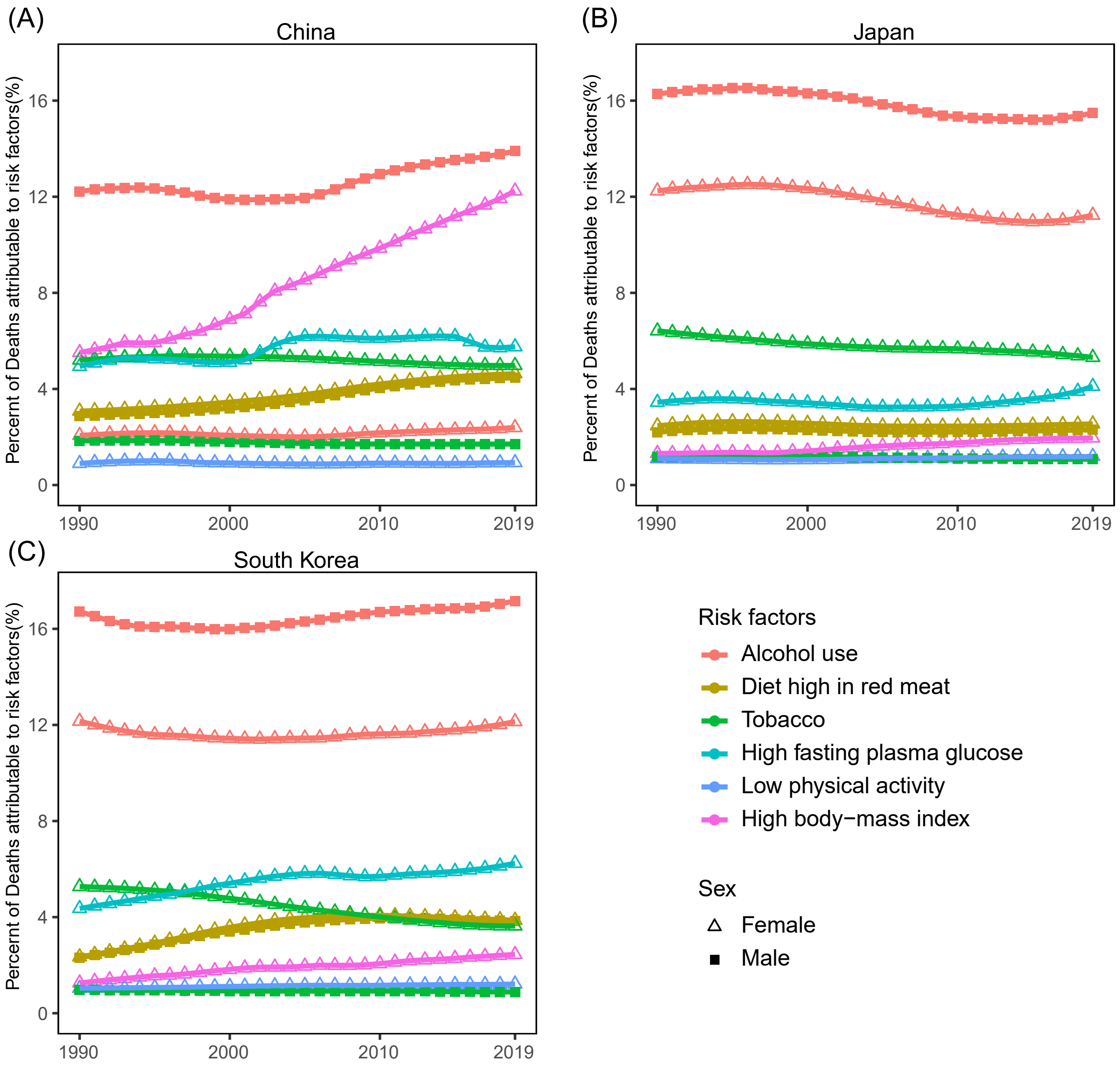
(A)
China
(B)
Japan
(C)
South Korea
Percent of Deaths attributable to risk factors(%)
0
4
8
12
16
1990
2000
2010
2019
Risk factors
Alcohol use
Diet high in red meat
Tobacco
High fasting plasma glucose
Low physical activity
High body−mass index
Sex
Female
Male

Figure S7

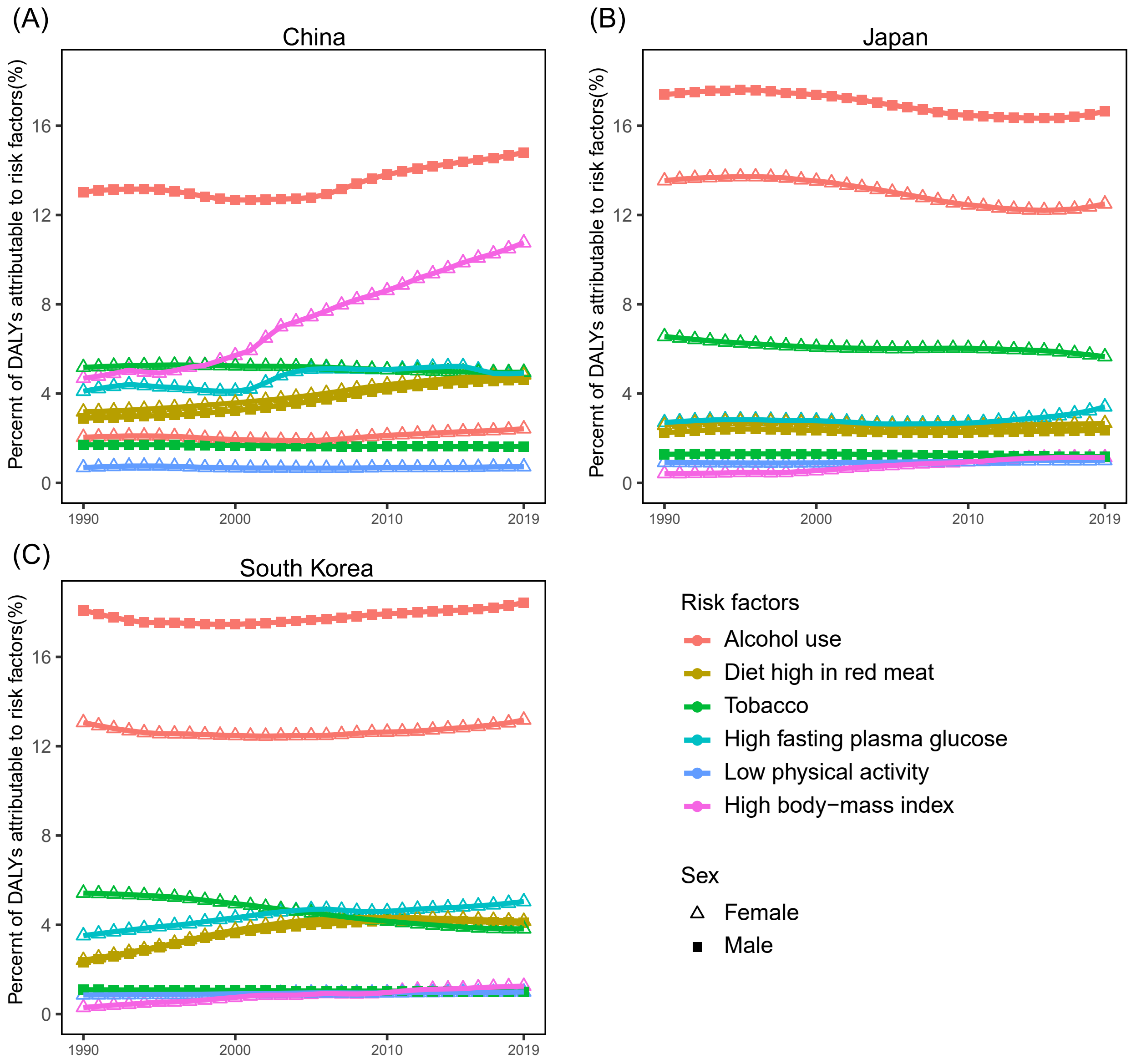
(A)
China
Percent of DALYs attributable to risk factors(%)
0
4
8
12
16
1990
2000
2010
2019
(B)
Japan
Percent of DALYs attributable to risk factors(%)
0
4
8
12
16
1990
2000
2010
2019
(C)
South Korea
Percent of DALYs attributable to risk factors(%)
0
4
8
12
16
1990
2000
2010
2019
Risk factors
Alcohol use
Diet high in red meat
Tobacco
High fasting plasma glucose
Low physical activity
High body−mass index
Sex
Female
Male

Figure S8

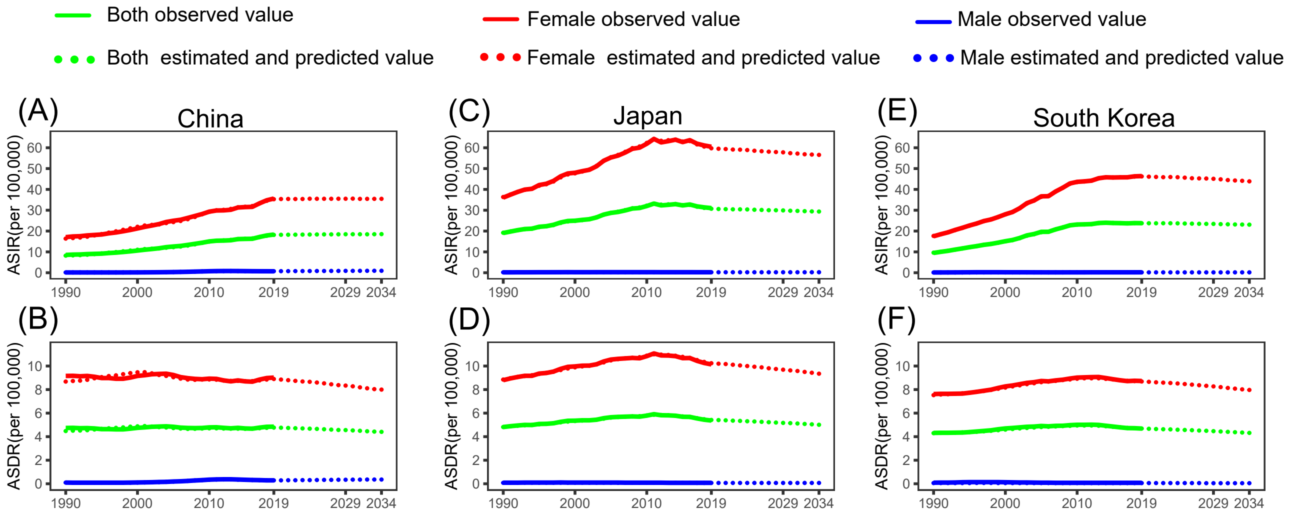
Both observed value
Female observed value
Male observed value
Both estimated and predicted value
Female estimated and predicted value
Male estimated and predicted value
(A)
China
(C)
Japan
(E)
South Korea
(B)
(D)
(F)
ASIR(per 100,000)
ASDR(per 100,000)
1990
2000
2010
2019
2029
2034

Figure S9

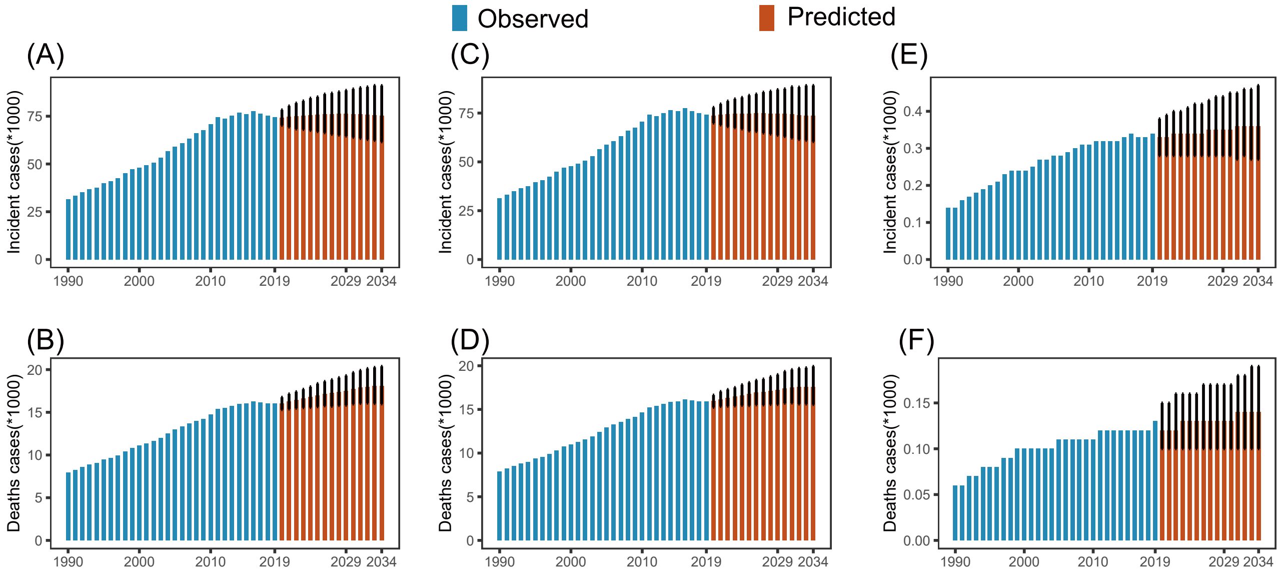
Observed
Predicted
(A)
Incident cases(*1000)
(B)
Deaths cases(*1000)
(C)
Incident cases(*1000)
(D)
Deaths cases(*1000)
(E)
Incident cases(*1000)
(F)
Deaths cases(*1000)
1990
2000
2010
2019
2029
2034

Figure S10

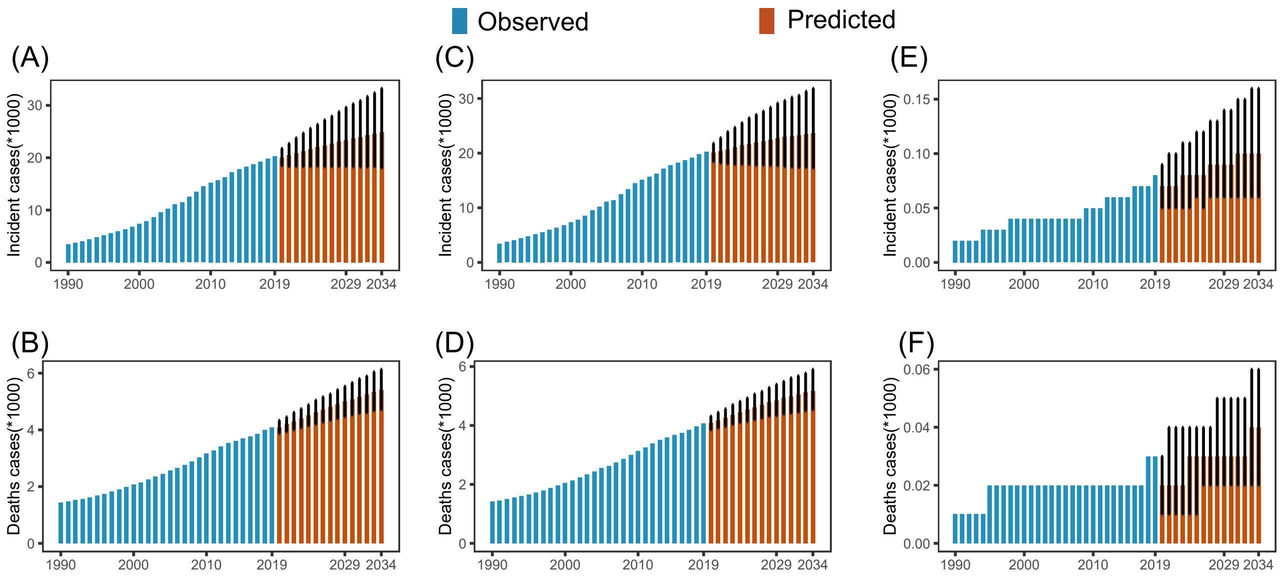
Observed
Predicted
(A)
(B)
(C)
(D)
(E)
(F)
Incident cases(*1000)
Deaths cases(*1000)
0
10
20
30
2
4
6
0.00
0.05
0.10
0.15
0.02
0.04
0.06
1990
2000
2010
2019
2029
2034
